# Disarming carbapenemase-producing *Acinetobacter baumannii*: high potency of the novel therapeutic combination of meropenem and the innovative diazabicyclooctane β-lactamase inhibitor pilabactam (formerly ANT3310)

**DOI:** 10.1128/aac.01691-25

**Published:** 2026-02-19

**Authors:** Salud Rodríguez-Pallares, Michelle Outeda-García, Emilio Lence, Arianna Rodríguez-Coello, Lucía González-Pinto, Paula Guijarro-Sánchez, Gabriela Alejandra Báez-Barroso, Tania Blanco-Martín, Juan Carlos Vázquez-Ucha, Agustina Llanos, Filomena Sannio, Jean-Denis Docquier, Ian Morrisey, Stephen Hawser, Magdalena Zalacain, Marc Lemonnier, Concepción González-Bello, Germán Bou, Alejandro Beceiro, Jorge Arca-Suárez

**Affiliations:** 1 Servicio de Microbiología Clínica & Grupo de Investigación en Microbiología, Instituto de Investigación Biomédica de A Coruña (INIBIC), Complexo Hospitalario Universitario de A Coruña (CHUAC), Sergas, Universidade da Coruña 16737, A Coruña, Spain; 2 CIBER de Enfermedades Infecciosas (CIBERINFEC), Instituto de Salud Carlos III 38176, Madrid, Spain; 3 Centro Singular de Investigación en Química Biolóxica e Materiais Moleculares (CiQUS), Departamento de Química Orgánica, Universidade de Santiago de Compostela 16780, Santiago de Compostela, Spain; 4 Departamento de Química Orgánica, Facultad de Ciencias, Universidad de Valladolid, Campus Miguel Delibes 88193 , Valladolid, Spain; 5 Antabio SAS, Labège, France; 6 Dipartimento di Biotecnologie Mediche, Università degli Studi di Siena 9313, Siena, Italy; 7 Antimicrobial Focus Ltd. 730898 , Sawbridgeworth, United Kingdom; 8 IHMA Europe, Monthey (Valais), Switzerland; 9 Department of Physiotherapy, Medicine and Biomedical Sciences, University of A Coruña 16737, A Coruña, Spain; University of Fribourg, Fribourg, Switzerland

**Keywords:** *Acinetobacter baumannii*, carbapenem resistance, ANT3310, pilabactam, OXA-23, β-lactamase, carbapenems, β-lactamase inhibitors

## Abstract

Carbapenem-resistant *Acinetobacter baumannii* (CRAB) represents an urgent global health threat, with resistance primarily driven by carbapenem-hydrolyzing class D β-lactamases (CHDLs) such as OXA-23. Therapeutic options remain limited due to the scarcity of effective β-lactam/β-lactamase inhibitor combinations. Pilabactam (formerly ANT3310) is a novel diazabicyclooctane (DBO) β-lactamase inhibitor featuring a fluorine substituent that extends its activity spectrum, relative to approved DBOs like avibactam and relebactam, to include CHDLs. Pilabactam is currently in phase I clinical trials in combination with meropenem, and its activity and mechanism against CRAB remain incompletely defined. Using engineered *A. baumannii* strains producing individual β-lactamases, we show that pilabactam restores meropenem activity against serine β-lactamase producers, including difficult-to-inhibit CHDLs. This was corroborated in 68 whole-genome-sequenced meropenem-resistant clinical isolates, yielding MIC₅₀ and MIC₉₀ values for meropenem/pilabactam of 1 and 2 mg/L, respectively. Frequency of resistance studies in representative CHDL producers demonstrated suppression of resistance selection at 4× MIC. Kinetic analyses revealed that pilabactam inhibits OXA-23 via a two-step tight binding mechanism, with slightly higher inactivation rates (1.7 × 10⁴ M⁻¹s⁻¹) than that of durlobactam (3.5 × 10³ M⁻¹s⁻¹). Pilabactam also yielded a low dissociation constant (*K_d_* ≈ 4 nM) and slow off-rate, indicating durable inhibition. Molecular dynamics simulations revealed the critical role of the fluorine substituent in forming stabilizing hydrogen-bonding and CH–F interactions within the tunnel-like OXA-23 active site. These findings identify pilabactam as a potent novel DBO supporting its development with meropenem for treating CRAB infections.

## INTRODUCTION

*Acinetobacter baumannii* is a major nosocomial pathogen frequently associated with life-threatening infections in immunocompromised or critically ill patients. *A. baumannii* infections are associated with high levels of morbidity and mortality, largely due to the scarcity of effective treatment options (1). The World Health Organization (WHO) has classified carbapenem-resistant *A. baumannii* (CRAB) as a critical priority pathogen, highlighting the urgent need for the discovery and development of new antibiotics (2). Broad-spectrum β-lactam resistance mechanisms in *A. baumannii* continue to evolve, becoming more diverse and prevalent. However, in most multidrug-resistant (MDR) and extensively drug-resistant (XDR) clinical isolates, carbapenem resistance is primarily driven by the horizontal acquisition of genes encoding carbapenem-hydrolyzing class D β-lactamases (CHDLs), particularly OXA-23, OXA-24/40, and OXA-58 (3). Beyond CHDLs, *A. baumannii* may further enhance β-lactam resistance through other multiple clinically relevant mechanisms, including overexpression of intrinsic *bla*_OXA-51-like_ or *bla*_ADC_ genes via upstream insertion of IS*Aba*1 elements, upregulation of efflux pump operons, modification of penicillin-binding protein (PBP) targets, and disruption of outer membrane proteins involved in β-lactam diffusion (4).

Recent β-lactam/β-lactamase inhibitor development has yielded agents pairing broad-spectrum β-lactams with one of two major classes of next-generation β-lactamase inhibitors: (i) 1,6-diazabicyclo[3.2.1]octane (DBO) derivatives (e.g., avibactam, relebactam, durlobactam) and (ii) cyclic boronates (e.g., vaborbactam) (5). Unfortunately, with the exception of durlobactam (formerly ETX2514), none of these inhibitors significantly enhances β-lactam activity against MDR or XDR *A. baumannii*, primarily due to poor outer membrane penetration and/or lack of inhibitory activity against CHDLs. Durlobactam differs structurally from avibactam because of the presence of an endocyclic double bond between the C3 and C4 positions and also the methyl group at C3, which confers improved potency through rapid acylation of both *Acinetobacter*-derived class C (ADC) and CHDLs. By protecting sulbactam from hydrolysis by CHDLs, the sulbactam/durlobactam (SUL/DUR) combination exhibits potent activity against CRAB isolates in most surveillance studies (6). However, the bactericidal activity of this combination primarily relies on sulbactam, which has a narrow PBP-binding spectrum and low acylation rates for PBP1 and PBP3 (7). Consequently, resistance is emerging among strains harboring alterations in the transpeptidase domain of these PBPs (8). Moreover, efflux also appears to play a role in modulating the activity of the combination, as evidenced by the lower minimum inhibitory concentrations (MICs) of SUL/DUR, but not sulbactam alone, for *A. baumannii* mutants lacking major RND efflux systems, such as AdeIJK and AdeABC, suggesting that durlobactam may act as a substrate for these pumps and that efflux contributes to resistance mechanisms (9, 10).

Cefiderocol is a novel β-lactam with potent activity against *A. baumannii*, combining cephalosporin-derived structural motifs for β-lactamase stability with a siderophore-mediated iron uptake mechanism that enhances outer membrane penetration (11, 12). However, its efficacy against *A. baumannii* is compromised by the increasing prevalence of β-lactamases with high cephalosporinase activity (e.g., PER-1) and the emergence of mutations in iron uptake systems during treatment (13, 14). Furthermore, the clinical use of cefiderocol for severe *A. baumannii* infections remains controversial, as all-cause mortality was numerically higher in patients treated with cefiderocol than in those receiving the best available therapy in clinical trials involving *Acinetobacter* spp. infections (15). In this context, the limited number of effective treatments for *A. baumannii* infections underscores the urgent need for the development of novel antimicrobial agents.

A novel breakthrough DBO-type β-lactamase inhibitor, pilabactam (formerly ANT3310), was recently discovered through the strategic replacement of the carboxamide group in avibactam with a fluorine atom. Distinct from previously developed DBOs, pilabactam exhibits potent inhibitory activity against all serine β-lactamases, with IC₅₀ values in the nanomolar range for Ambler class A, C, and D enzymes (16, 17) and does not have intrinsic antibacterial activity (18). In combination with meropenem, pilabactam has demonstrated promising *in vitro* activity against serine β-lactamase-producing Gram-negative bacteria, as well as *in vivo* efficacy in murine infection models, including those involving CHDL-producing *A. baumannii* (17, 18). The first-in-human study of the meropenem/pilabactam (MEM/PIL) combination (NCT05905913) has been successfully completed; the combination is currently undergoing a phase 1 pharmacokinetic study in subjects with various degrees of renal function impairment (NCT06527677) and in healthy subjects in a lung penetration study (NCT06916156). The MEM/PIL combination may thus represent a significant therapeutic advancement for the treatment of severe *A. baumannii* infections. However, beyond these initial observations and limited *in vitro* findings based on randomly selected surveillance isolates, the mechanistic basis for the high potency of MEM/PIL against *A. baumannii* remains poorly understood. Building on our previous work on the epidemiology, resistance mechanisms in *A. baumannii*, and novel CHDL β-lactamase inhibitors (19–22), we sought to elucidate the molecular basis of the remarkable inhibitory activity of pilabactam, with particular emphasis on its interaction with CHDLs. Our findings have important medical implications, as they provide novel insights into the mechanism of action of pilabactam against class D β-lactamases and support the therapeutic positioning of the MEM/PIL combination for the treatment of carbapenem-resistant *A. baumannii* infections, which poses a major clinical challenge.

## RESULTS AND DISCUSSION

### MEM/PIL exhibits potent activity against *A. baumannii* recombinant strains producing serine β-lactamases

To assess the potency and spectrum of β-lactamase inhibition by pilabactam in *A. baumannii*, we evaluated the activity of MEM/PIL against 15 isogenic transformants producing distinct, clinically relevant β-lactamases. Using meropenem as a reporter antibiotic, the potentiation effect of pilabactam was also compared with that of durlobactam, tested at 4 mg/L, the standard concentration for this compound, and at 8 mg/L, matching the testing concentration of pilabactam to enable direct comparison. The activities of cefiderocol, sulbactam alone, and SUL/DUR were also evaluated. Comparative MIC data for the transformants are summarized in Table 1. As shown, expression of β-lactamase genes with carbapenemase activity in the *A. baumannii* ATCC 17978 background increased the meropenem MICs from 0.25 mg/L to between 4 and >64 mg/L, confirming the suitability of our experimental model. Meropenem retained activity (with only modest increases in MIC) against transformants producing extended-spectrum β-lactamases (ESBLs) lacking carbapenemase activity, such as GES-1 (23), CTX-M-15 (24), SHV-12 (25), and PER-1 (26), all previously identified in clinical *A. baumannii* isolates. Sulbactam was more strongly affected by these ESBLs, but less so by CHDL enzymes. As expected, cefiderocol displayed the highest activity but was to some extent affected by the production of PER, SHV, and NDM-type enzymes.

**TABLE 1:** Antibiotic susceptibility data for the *A. baumannii*-derived recombinant isolates producing class A, B, and D β-lactamases against β-lactam/β-lactamase inhibitor combinations and cefiderocol^*e*^

Strain	Ambler class	MIC (mg/L)						
		MEM (R ≥ 8)^*a*^	M/P (8 mg/L)^*b*^ (R ≥ 8)^*c*^	M/D (4 mg/L)^*b*^ (R ≥ 8)^*c*^	M/D (8 mg/L)^*b*^ (R ≥ 8)^*c*^	SUL (R ≥ 16)^*d*^	S/D (4 mg/L)^*b*^ (R ≥ 16)^*a*^	FDC (R ≥ 16)^*a*^
*A. baumannii* ATCC 17978	–	0.25	≤0.06	0.125	0.125	0.5	0.5	≤0.06
GES-1	A	0.5	0.125	0.125	0.125	8	0.5	≤0.06
GES-5	A	4	0.125	0.125	0.125	4	0.5	0.125
CTX-M-15	A	0.5	0.25	0.125	0.125	8	0.5	0.25
SHV-12	A	0.5	0.125	0.125	0.125	4	0.5	1
PER-1	A	0.5	0.25	0.125	0.125	16	0.5	16
TEM-52	A	2	1	1	1	8	2	0.25
KPC-3	A	32	0.125	0.125	0.125	32	0.5	0.125
IMP-2	B	32	16	16	16	2	1	0.25
NDM-1	B	32	32	32	16	8	4	0.5
OXA-23	D	8	0.125	0.5	0.5	2	1	≤0.06
OXA-24/40	D	64	0.25	0.25	0.25	2	0.5	≤0.06
OXA-51	D	4	0.125	0.125	0.125	2	0.5	≤0.06
OXA-58	D	8	0.125	0.125	0.125	8	0.5	≤0.06
OXA-143	D	>64	0.125	0.5	0.5	2	0.5	≤0.06
OXA-235	D	4	0.125	0.125	0.125	2	1	≤0.06

^*a*^ 2025 CLSI breakpoint indicated.

^*b*^ Concentration at which the β-lactamase inhibitor was added.

^*c*^ For M/P and M/D, a breakpoint of 8 mg/L was applied.

^*d*^ For SUL, a breakpoint of 16 mg/L was applied.

^*e*^ MEM, meropenem; M/P, meropenem/pilabactam; M/D, meropenem/durlobactam; SUL, sulbactam; S/D, sulbactam/durlobactam; FDC, cefiderocol. –, not applicable.

MEM/PIL demonstrated potent activity against all transformants producing serine β-lactamases, with MICs consistently within the susceptibility range and not exceeding 1 mg/L. In fact, the majority of transformants yielded MICs between 0.125 and 0.25 mg/L. The most notable MIC reductions were observed in transformants producing KPC-3 (from 32 to 0.125 mg/L; 256-fold decrease), OXA-24/40 (from 64 to 0.25 mg/L; 256-fold) and OXA-143 (from >64 to 0.125 mg/L; >512-fold). The ability of pilabactam to restore meropenem activity was comparable to that of durlobactam, at both 4 and 8 mg/L, indicating that these structurally distinct DBO-type inhibitors provide a similar spectrum of activity and *in vitro* potency against *A. baumannii* strains harboring different serine-type β-lactamases.

Data obtained from these isogenic isolates provide valuable insights into the comparative activity of MEM/PIL and SUL/DUR against *A. baumannii*: (i) in the absence of CHDLs, meropenem exhibits intrinsically higher antibacterial activity than sulbactam. This previously reported phenomenon (7) is attributed to the broader binding spectrum of meropenem to PBPs and higher acylation rates against key transpeptidases (PBP-1, PBP-2, and PBP-3) in *A. baumannii*; (ii) meropenem and sulbactam are differently impacted by the β-lactamases commonly found in *A. baumannii*: meropenem is more susceptible to degradation by carbapenemases, whereas sulbactam is more affected by β-lactamases with ESBL activity; (iii) both pilabactam and durlobactam effectively restore the susceptibility of *A. baumannii* strains producing serine β-lactamases, as evidenced by the comparable performance when combined with the same β-lactam agent. These findings help explain the slightly higher activity observed for the MEM/PIL combination.

### Pilabactam overcomes meropenem resistance in clinical *A. baumannii* strains producing CHDLs

Sixty-eight representative whole-genome-sequenced CRAB isolates from the 2020 Spanish nationwide surveillance survey were tested for susceptibility to meropenem with and without pilabactam. For comparative purposes, cefiderocol, sulbactam, and SUL/DUR were also evaluated. Most isolates belonged to the globally disseminated ST2 (*n* = 52; 76.5%) and ST1 (*n* = 11; 16.2%) clones. Less prevalent sequence types included ST745 (*n* = 4; 5.9%), ST85 (*n* = 2; 2.9%) and ST25 (*n* = 1; 1.5%). In most isolates, carbapenem resistance was associated with production of CHDLs such as OXA-23 (*n* = 49), OXA-58 (*n* = 8), and OXA-24/40 (*n* = 1). IS*Aba*1 was identified in 10 isolates upstream of *bla*_OXA-51-like_ genes, which is known to provide strong promoter sequences that enhance the expression of intrinsic β-lactamase genes in this species (27). These insertion sequences were also found in some isolates upstream of *bla*_ADC_ genes. Among OXA-51-like variants, OXA-66 was the most frequently identified (*n* = 45; 66.2%), followed by OXA-69 (*n* = 10; 14.7%), OXA-201 (*n* = 10; 14.7%), OXA-94 (*n* = 2; 2.9%), and OXA-64 (*n* = 1; 1.5%), whereas the ADC-like cephalosporinases exhibited greater genetic diversity, including ADC-30 (*n* = 55; 80.9%), ADC-75 (*n* = 10; 14.7%), ADC-2 (*n* = 2; 2.9%), and ADC-5 (*n* = 1; 1.5%). Other notable β-lactam resistance-associated alterations included disruptions in outer membrane proteins such as Omp33-36 (*n* = 6), OprD (*n* = 2), CarO (*n* = 1), and Omp25 (*n* = 1). A detailed perspective of antimicrobial susceptibility data for the agents evaluated alongside the resistome of each strain analyzed is available in Table S1.

**Fig 1:**
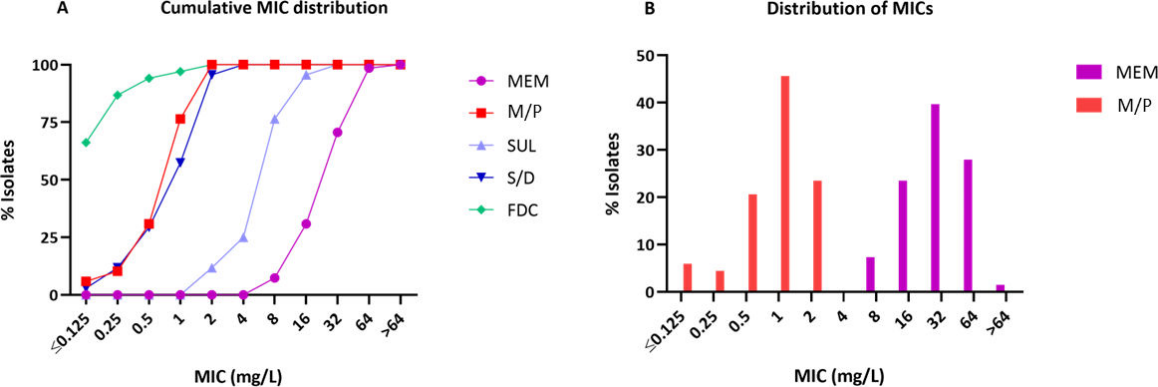
(**A**) Cumulative MIC distribution of meropenem (MEM), meropenem/pilabactam (M/P), sulbactam (SUL), sulbactam/durlobactam (S/D), and cefiderocol (FDC) against *A. baumannii* carbapenem-resistant clinical isolates. (**B**) Distribution of MEM and M/P MICs for the whole collection of clinical isolates.

The cumulative MIC distribution for meropenem, MEM/PIL, and comparators for the entire collection is presented in Fig. 1A. The MIC₅₀ and MIC₉₀ values, the range of MICs, and susceptibility percentages, both for the overall collection and for representative subgroups, are further detailed in Table 2. As expected for carbapenem-resistant strains, all isolates yielded meropenem MICs ranging from 8 to 64 mg/L, with MIC₅₀ and MIC₉₀ values of 32 and 64 mg/L, respectively. By contrast, the MEM/PIL combination demonstrated markedly enhanced activity, with MIC₅₀ and MIC₉₀ values of 1 and 2 mg/L, respectively. All isolates were inhibited at ≤2 mg/L, corresponding to 100% susceptibility when applying a susceptibility breakpoint of 8 mg/L (Fig. 1B). These findings indicate that pilabactam enhances meropenem activity against CRAB by up to 32-fold. These experimental data are consistent with recent results from 703 CRAB surveillance isolates collected in 2018–2019 within the IHMA global collection, which reported MIC₅₀ and MIC₉₀ values of 1 and 8 mg/L, respectively (18).

**TABLE 2:** Cumulative MIC distribution, MIC_50_, MIC_90_, range, and percentage of susceptible and resistant isolates for β-lactam/β-lactamase inhibitor combinations and cefiderocol against carbapenem-resistant *A. baumannii* clinical isolates^*c*^

Genotype features	Antimicrobial agent^*a*^	Cumulative % inhibited at MIC (mg/L) of											MIC_50_	MIC_90_	Range	% Sus^*b*^	% Res^*b*^
		≤0.125	0.25	0.5	1	2	4	8	16	32	64	>64					
OXA-23 (*N* = 49)	MEM							2.04	8.16	61.22	100		32	64	8-64	0	100
	M/P	6.12	8.16	26.53	77.55	100							1	2	≤0.125–2	100	0
	SUL						10.2	75.51	93.88	100			8	16	4–32	75.51	24.29
	S/D	2.04	8.16	30.61	59.18	93.88	100						1	2	≤0.125–4	100	0
	FDC	71.43	87.76	93.88	95.92	100							≤0.125	0.5	≤0.125–2	100	0
OXA-58 (*N* = 8)	MEM							50	100				8	16	8–16	0	100
	M/P		12.5	50	87.5	100							0.5	2	0.25–2	100	0
	SUL						25	62.5	100				8	16	4–16	62.5	37.5
	S/D	12.5	50	62.5	100								0.25	1	≤0.125–1	100	0
	FDC	62.5	87.5	100									≤0.125	0.5	≤0.125–0.5	100	0
IS*Aba*1-OXA-51-like (*N* = 10)	MEM								90	100			16	16	16–32	0	100
	M/P	20	10	30	60	100							1	2	≤0.125–2	100	0
	SUL					80	100						2	4	2–4	100	0
	S/D				10	100							2	2	1–2	100	0
	FDC	40	80	90	100								0.25	0.5	≤0.125–1	100	0
All isolates (*N* = 68)	MEM							7.35	30.88	72.06	98.53	100	32	64	8 to >64	0	100
	M/P	10.94	9.38	31.25	75.0	100							1	2	≤0.125–2	100	0
	SUL					11.76	25.0	76.47	95.59	100			8	16	2–32	76.47	23.53
	S/D	2.94	11.76	29.41	57.35	95.59	100						1	2	≤0.125–4	100	0
	FDC	66.18	86.76	94.11	97.06	100							≤0.125	0.5	≤0.125–2	100	0

^*a*^ Pilabactam was tested at a fixed concentration of 8 mg/L. Durlobactam was tested at a fixed concentration of 4 mg/L.

^*b*^ Sus, susceptible; Res, resistant. The 2025 CLSI breakpoint was applied for MEM, S/D, and FDC. For M/P, a breakpoint of 8 mg/L was applied. For SUL, a breakpoint of 16 mg/L was applied.

^*c*^ MEM, meropenem; M/P, meropenem/pilabactam; SUL, sulbactam; S/D, sulbactam/durlobactam; FDC, cefiderocol.

SUL/DUR also demonstrated remarkable activity, achieving 100% susceptibility with MIC₅₀ and MIC₉₀ values of 1 and 2 mg/L, respectively (identical to those observed for MEM/PIL), and showing no significant variation across the subgroups evaluated. These MIC values are consistent with those previously reported for CRAB surveillance isolates from hospitals in the US (28). Closer analysis of the SUL/DUR MICs revealed that the potentiating effect of durlobactam on sulbactam, reflected in the reduction of MIC₅₀ and MIC₉₀ from 8 and 16 mg/L to 1 and 2 mg/L, respectively, is less pronounced (an 8-fold decrease) than the effect observed after the addition of pilabactam to meropenem. These findings are consistent with previous observations for the isogenic strains and highlight the key role of the intrinsic stability of sulbactam in the potent activity of the SUL/DUR combination against CHDL-producing *A. baumannii* isolates. Indeed, sulbactam alone inhibited 76.47% of the tested isolates at 8 mg/L, whereas meropenem at the same concentration inhibited only 7.35%. Cefiderocol was the most active β-lactam against this collection; however, its clinical application for *A. baumannii* infections remains a subject of debate due to the lack of translation between low *in vitro* MICs and clinical efficacy, as highlighted by the findings of the CREDIBLE study (15). Although MBL-producing *A. baumannii* isolates, particularly those harboring NDM, are increasingly reported worldwide (29), none were identified in this surveillance study. However, based on our data for isogenic isolates, as well as findings reported by other researchers, most of the β-lactams evaluated in this study are not expected to retain significant activity against the metallo-carbapenemase producers (30). On the other hand, the inclusion of SUL/DUR-resistant isolates, especially those carrying PBP modifications or loss-of-function mutations leading to efflux pump upregulation, would be of particular interest, as this would enable a more thorough assessment of the stability and efficacy of MEM/PIL against strains harboring these emerging resistance mechanisms.

### MEM/PIL shows a low propensity of development of resistance

The frequency of spontaneous resistance (FoR) and mutant prevention concentrations (MPCs) in four representative carbapenem-resistant, CHDL-producing *A. baumannii* clinical isolates is summarized in Table S2. These isolates carried either OXA-23 (3/4 strains) or OXA-24/40 (1/4 strains) carbapenemases and yielded meropenem agar MICs of 64, 64, 64, and 256 mg/L, respectively. Upon addition of pilabactam, the meropenem MICs were reduced to 2, 4, 1, and 1 mg/L, respectively. No mutants resistant to MEM/PIL were selected at concentrations corresponding to 4× the MIC values with any of these strains, thereby resulting in FoR rates of <1.2 × 10^−9^. Consequently, the MPC values for all strains were determined to be ≤4× their respective MEM/PIL MIC, ranging in all cases from 4 to 16 mg/L. Similar studies performed with the SUL/DUR combination at 4× the MIC reported FoR rates in *A. baumannii* strains below 7.6 × 10^−10^ (31). The suppression of the emergence of MEM/PIL-resistant mutants observed in these experiments is encouraging, suggesting that the potential for resistance development of MEM/PIL during treatment of CRAB infections is low, supporting further clinical development of the combination. Ongoing investigations using hollow-fiber infection models to more precisely determine the levels of exposure that prevent the emergence of resistance, murine models with humanized dosing, and upcoming human PK/PD data from phase 1 studies are expected to provide valuable insights for optimizing MEM/PIL dosing strategies against *A. baumannii*.

### Kinetic characterization of pilabactam and durlobactam against the OXA-23 CHDL

To perform a detailed comparative analysis of the inhibitory potency of pilabactam and benchmark its performance against other developed DBOs, the OXA-23 CHDL was purified, and its inactivation by pilabactam and durlobactam was extensively studied using enzyme kinetic methods. Prior to determining inhibitory parameters, the hydrolysis rates of nitrocefin, used as the reporter substrate, by OXA-23 were measured. These catalytic constants are consistent with previously reported values for OXA-23 by our group and other authors (19, 32). Thus, demonstrating adequate enzymatic activity to perform kinetics assays.

The β-lactamase inhibitors pilabactam and durlobactam exhibited comparable inhibition kinetics against OXA-23, characterized by high second-order rate constants and low dissociation constants, indicating strong and sustained enzyme-inhibitor interactions. These results point to a fast and efficient acylation process and a very stable covalent adduct between OXA-23 and pilabactam. Altogether, these results clearly demonstrate that pilabactam is a novel DBO-type β-lactamase inhibitor with potent activity against the OXA-23 CHDL. This is particularly relevant given the extremely limited therapeutic solutions targeting CHDL-producing CRAB, considering both commercially available (cefiderocol, SUL/DUR) and under clinical development options (such as xeruborbactam combined with meropenem or cefiderocol). Such combinations include other β-lactamase inhibitors reported to have activity against CHDLs, such as xeruborbactam (showing *K_i app_* values in the nanomolar range) (33), funobactam, or pralurbactam, although their specific activity against OXA-23 has not yet been disclosed, and none of them have demonstrated clinically relevant coverage of CRAB (34, 35).

A closer look at the mechanism of β-lactamase inhibition allowed us to identify differences in the second order-rate constants of pilabactam- and durlobactam-mediated OXA-23 inactivation, suggesting distinct kinetic behaviors. In this regard, pilabactam showed a hyperbolic *k_obs_* vs inhibitor concentration ([I]) plot, consistent with a two-step, slow-tight binding mechanism leading to formation of a covalent enzyme-inhibitor complex, while durlobactam showed a linear *k_obs_* vs [I] plot under our experimental conditions. This suggests that the formation or stability of the Michaelis complex (initial enzyme-inhibitor complex) with pilabactam is faster or more favorable than with durlobactam. Durlobactam binds to and/or stabilizes the initial complex with OXA-23 less effectively than pilabactam, in a manner similar to what is observed with avibactam (36, 37). Nevertheless, both inhibitors are expected to follow a two-step reversible acylation mechanism, and the observed differences likely reflect variations in the rate constants governing Michaelis complex formation and covalent adduct formation, as well as the potential reversibility of the latter step.

With this in mind, the second-order rate constants *k_inact_*/*K_I_* and *k*_+2_/*K* provide a comprehensive basis for comparing the overall inhibitory efficiencies of these compounds. The DBO pilabactam demonstrated a high inhibitory efficiency (*k_inact_*/*K_I_*: 16.9 × 10^3^ M^−1^ s^−1^) (Table 3), significantly outperforming avibactam, which showed an inhibitory efficiency approximately 60 times lower (*k_inact_*/*K_I_*: 2.9 × 10^2^ M^−1^ s^−1^) based on previously reported values using the same methodology (19). Moreover, durlobactam also exhibits substantial inhibitory efficiency, with a *k*_+2_/*K* value of 3.5 × 10^3^ M^−1^ s^−1^ (Table 3), concordant to that reported previously (5.1 ± 0.2 × 10^3^ M^−1^ s^−1^) (38).

**TABLE 3:** Kinetic parameters for OXA-23 and β-lactamase inhibitors^*b*^

β-lactamase inhibitors	*k*_+2_/*K^a^* (M^−1^ s^−1^)	*k_inact_* (s^−1^)	*K_I_* (μM)	*t*_n_	*k*_off_ (s^−1^)	*t_1/2_* (min)	*K_d_* (nM)
Pilabactam	(16.9 ± 3.2) × 10^3^	(6.6 ± 0.78) × 10^−3^	0.39 ± 0.06	60	(6.9 ± 1.41) × 10^−5^	171 ± 35	4.1 ± 1.04
Durlobactam	(3.5 ± 0.4) × 10^3^	–	–	2	(3.4 ± 0.013) × 10^−5^	337 ± 1.3	9.9 ± 1.1

^*a*^ Inhibitor efficiencies reflected as* k_inact_/K_I _*for pilabactam and as *k*_+2_/*K *for durlobactam.

^*b*^ Data represents the means of three independent experiments. –, not determined.

Additionally, the partition ratio (*t*_n_) values provided additional evidence of inhibitor efficiency, representing the stoichiometric relationship between substrate turnover and enzyme inactivation. This parameter correlated well with the previously described kinetic data. Notably, durlobactam exhibited a significantly lower I:E ratio of 2, compared to 60 for pilabactam (Table 3) and 150 for avibactam, as previously reported (19), indicating that durlobactam achieves almost complete inhibition of OXA-23 at lower concentrations.

Finally, both inhibitors exhibited low equilibrium dissociation constant (*K_d_*) values in the nanomolar range, with pilabactam showing approximately a 2-fold lower *K_d_* value (4.1 nM) than durlobactam (9.9 nM) (Table 3). The dissociation rate constant (*k_off_*), which represents the rate at which the enzyme-inhibitor complex dissociates, was notably low for pilabactam at 6.9 × 10^−5^ s⁻¹, similar to the *k_off_* of durlobactam at 3.4 × 10^−5^ s⁻¹; these findings are consistent with recent results determining the *k_off_* of durlobactam against other CHDLs, including OXA-58 enzyme (9.6 × 10^−3^ min^−1^) and OXA 24/40 (1.0 × 10^−3^ min^−1^) (6, 38). In accordance with these *k_off_* values, the residence times (*t*_1/2_) for pilabactam and durlobactam against OXA-23 were 171 and 337 minutes, respectively, indicating a significantly slow recovery of β-lactamase activity following inhibition (Table 3). Altogether, these data demonstrate that pilabactam effectively and nearly irreversibly inactivates OXA-23 through a combination of high binding affinity and carbamoylation rates, and slow dissociation. Both pilabactam and durlobactam form a very stable covalent adduct, but the reactivity of pilabactam is significantly higher than that of durlobactam, as reflected by the 5-fold higher rate of formation of such a covalent adduct. This potentially relies on a better early recognition of the inhibitor with the enzyme, leading to the formation of the Michaelis complex, or the stability of this complex itself, aspects that were investigated with molecular dynamics simulations experiments, as shown below.

To understand if this behavior, i.e., a better reactivity of pilabactam toward OXA-23, especially when compared to avibactam, could apply to other relevant CHDLS, a similar kinetic analysis was also performed with the OXA-24/40 enzyme. As a result, the measured rates for the formation of the covalent adduct (*k*_+2_/*K*) were 1.2 × 10^3^ and 58 M^−1^ s^−1^ with pilabactam and avibactam, respectively. The significantly (≈ 20-fold) higher rate of formation of the OXA-24/40 covalent adduct with pilabactam, when compared to that measured with avibactam, is also similar to that observed with OXA-23 (*k*_+2_/*K* value ≈ 15-fold higher for pilabactam). Overall, these data show that pilabactam inactivates these two CHDLs much faster than avibactam, through either a better recognition of the enzyme (formation of the Michaelis complex) or a better reactivity of the Michaelis complex itself, or both.

### *In silico* binding mode analysis reveals key differences in pilabactam and durlobactam interactions with OXA-23

**Fig 2:**
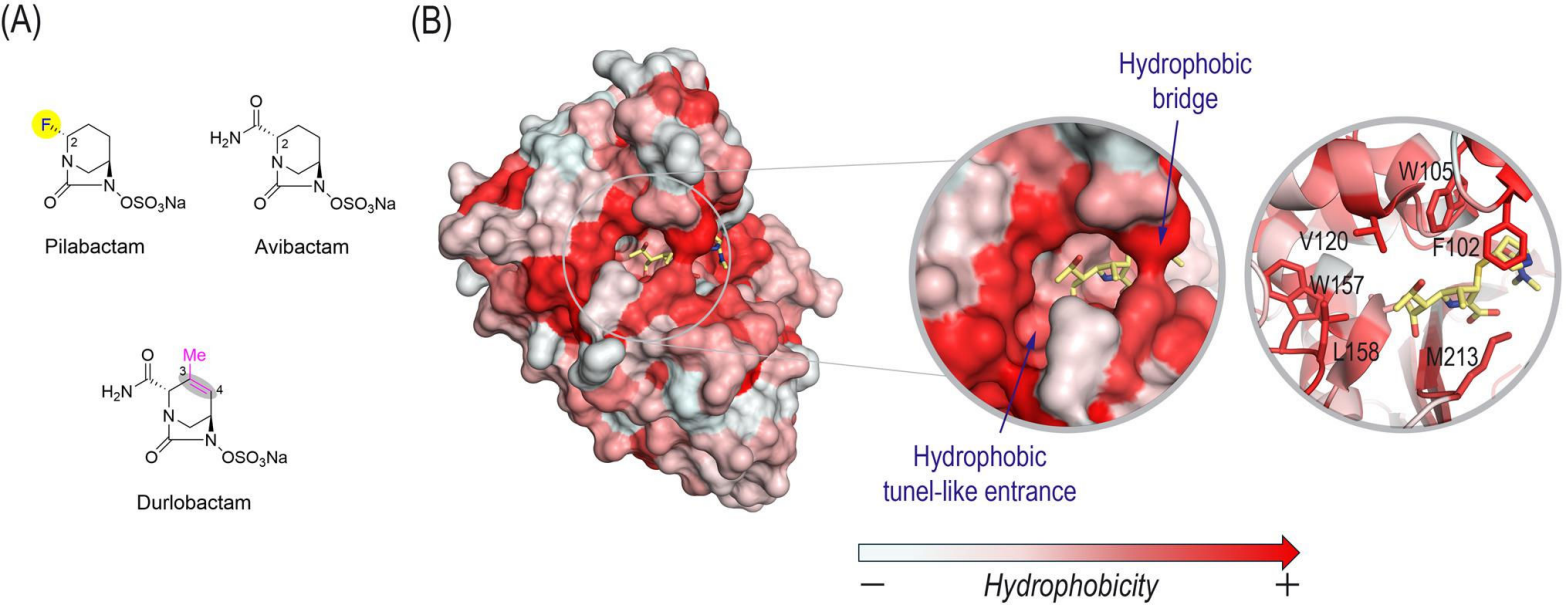
(**A**) Chemical structures of pilabactam, avibactam, and durlobactam. The main structural differences relative to avibactam are highlighted. (**B**) Crystal structure of OXA-23 from *A. baumannii* covalently bound to meropenem (PDB ID 4JF4, 2.14 Å). The hydrophobic surface and the uncommon tunnel-like entrance architecture are shown. Relevant side chain residues involving the hydrophobic tunnel-like entrance and the active site bridge are shown and labeled. The SAND consensus numbering for class D enzymes was used.

To gain a deeper understanding of the molecular basis underlying the strong inhibitory activity of pilabactam against the OXA-23 enzyme, the binding mode was investigated *in silico* and compared with that of durlobactam. As pilabactam is derived from avibactam, the first-in-class DBO inhibitor, by replacing the primary carboxamide moiety with a small electron-withdrawing substituent (a fluorine atom), but without the conformational rigidification of the bicyclic DBO core observed in durlobactam, the binding mode of avibactam was also examined, for comparative purposes (Fig. 2A). The proposed binding modes were obtained by docking, implemented with the GOLD program (version 2022.3.01) (39) and using the enzyme coordinates from the crystal structure of OXA-23 from *A. baumannii* covalently modified by meropenem (PDB ID 4JF4 [40], 2.14 Å resolution). The docking results were further refined and explored through molecular dynamics (MD) simulations with AMBER, providing a more realistic depiction of the conformational arrangement of DBO on binding. MD studies were conducted on both the OXA-23/inhibitor enzyme Michaelis complexes, which are formed prior to the covalent modification of the β-lactamase (resulting in an acyl-enzyme adduct representing the inactivated enzyme form). To this end, the GOLD-derived enzyme-inhibitor complexes were solvated in a truncated octahedron box of TIP3P water molecules and neutralized by adding sodium ions. The systems were parameterized using the ff14SB (41) force field of AMBER 21 and subjected to 100 ns of dynamic simulation, following our previously reported protocols (42, 43).

Like all serine β-lactamases, OXA-23 contains three conserved motifs: STFK (positions 70–73), SAV (positions 118–120), and KTG (positions 208–210). More importantly, the three-dimensional structure of OXA-23-like CHDLs is distinguished by an uncommon active site architecture, which except for OXA-24/40 variants, is markedly different from that of other β-lactamases (Fig. 2B). Specifically, the active site forms a highly hydrophobic, tunnel-like structure, primarily composed of the side chains of residues M213 and F102. This tunnel controls the positioning of carbapenems in a productive conformation for an extended period (44). Our molecular dynamics simulation studies of the wild-type enzyme reveal that this hydrophobic “bridge” remains conformationally stable and does not undergo significant changes (Fig. S1). Another notable feature is the large hydrophobic region adjacent to the tunnel-like active site entrance, mainly involving residues W105, V120, W157, and L158 (the latter two of which are involved in the Ω-loop) (Fig. 2B). These structural characteristics create a hydrophobic filter that selectively controls substrate access, largely explaining the ability of OXA-23 to hydrolyze carbapenems and its role as the predominant carbapenemase in *Acinetobacter* species (44).

**Fig 3:**
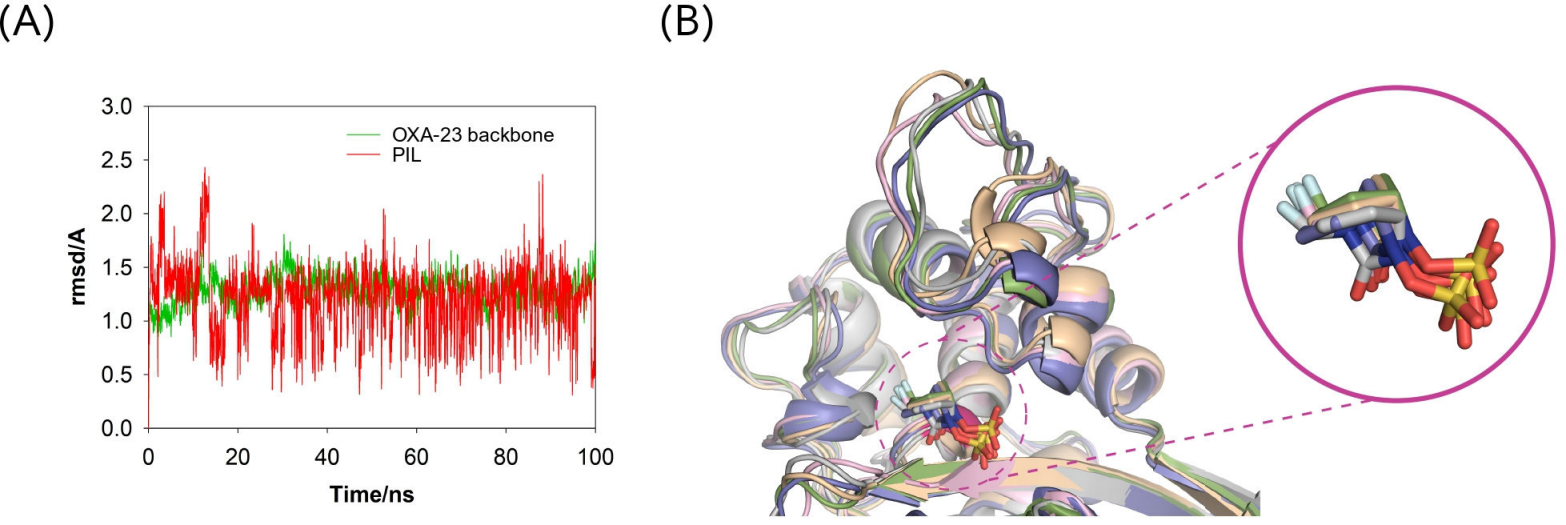
(**A**) RMSD plots for the enzyme backbone (Cα, C, O, and N atoms) and the ligand calculated from the 100 ns simulation of the OXA-23/pilabactam enzyme complex. Average RMSD values of 1.3 and 1.2 Å were obtained for the enzyme backbone and pilabactam, respectively. (**B**) Superposition of several snapshots from the 100 ns of MD simulation on the OXA-23/pilabactam enzyme complex. Note how pilabactam is well anchored in the active site since its position barely fluctuated during the whole simulation.

We found that all MD simulations performed using the catalytic lysine residue K73 in its *N*-carboxylated form, typically observed in all OXA enzymes (45), resulted in low stability of the DBO inhibitors within the active site, leading to their early displacement from the catalytic pocket. By contrast, when K73 was modeled in its neutral (non-carboxylated) form in the corresponding Michaelis complexes, the DBO inhibitors remained stably positioned within the active site throughout the entire 100-ns simulation, consistently located close to the catalytic serine residue S70. This observation is supported by the low root-mean-square deviation (RMSD) values obtained for both the ligands and the enzyme during the simulation period (Fig. 3). These results suggest that effective binding of the inhibitors may be associated with the *N*-decarboxylation of residue K73, a phenomenon also identified previously in the crystal structure of OXA-48 inactivated by pilabactam (PDB ID 6ZXI, 1.85 Å) (16). Although the *N*-carboxylated form of K73 is essential for *in vivo* enzymatic activity, its decarboxylation has been shown to hinder efficient hydrolysis of the acyl-enzyme intermediate, thereby preventing catalytic turnover and contributing to sustained enzyme inhibition (45).

**Fig 4:**
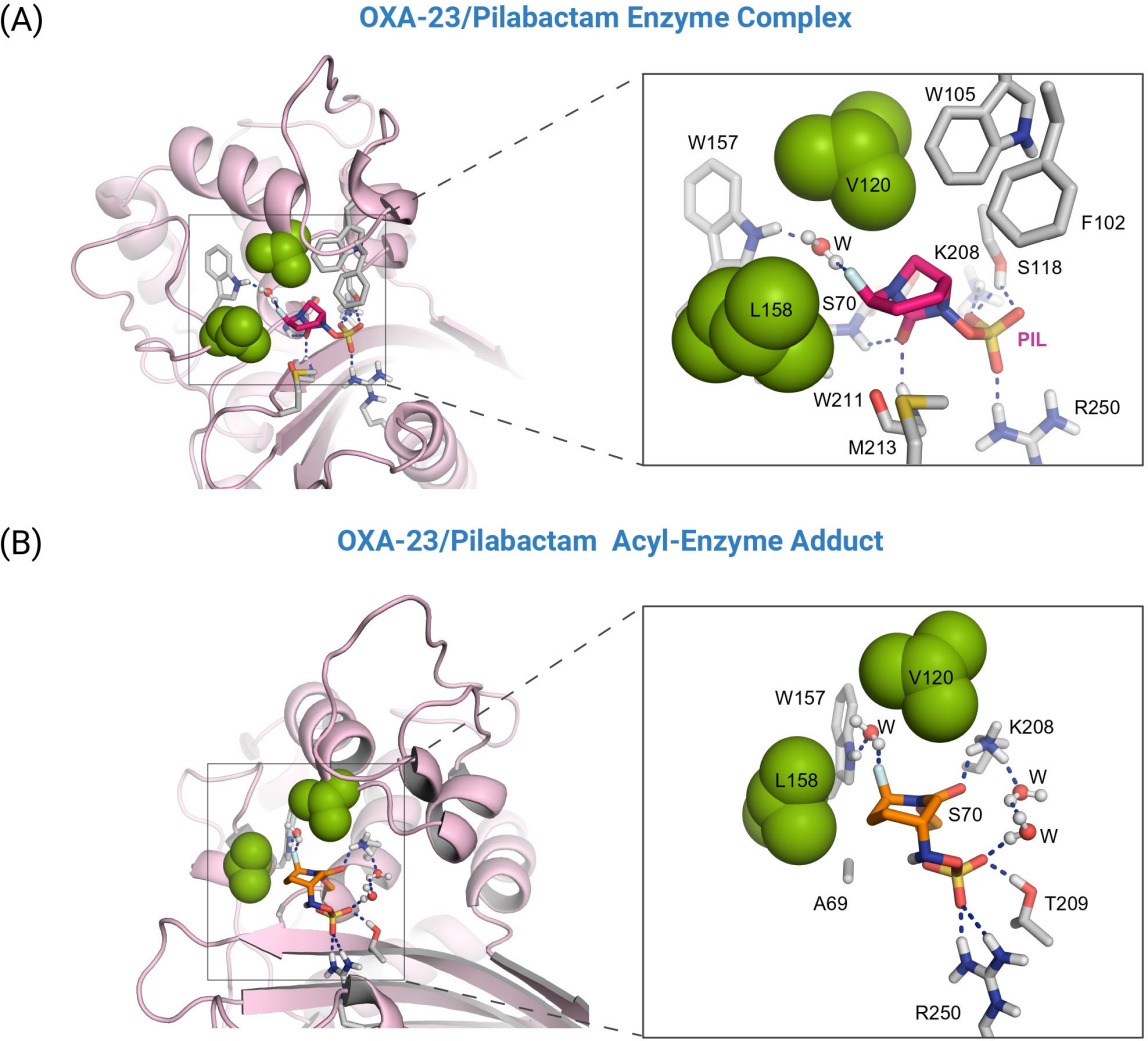
Proposed three-dimensional models of the OXA-23/pilabactam enzyme complex (**A**) and the corresponding enzyme adduct (**B**) obtained by MD simulation studies. Both an overall and a close-up view are shown. Snapshots were taken after 100 ns of dynamic simulation. Relevant side-chain residues (gray) are shown and labeled. Key hydrogen-bonding interactions are represented by dashed blue lines. Pilabactam is highlighted in pink (panel A), and the modified residue S70 (panel B) is shown in orange. Note how the fluorine atom would establish a strong hydrogen bond with residue W157 through a water molecule, which remains trapped in this position throughout the whole simulation and is isolated from the environment by residues L158 and V120 (green spheres). The latter interaction is enabled by the precise alignment of the functional groups involved.

For the OXA-23/pilabactam enzyme complex, the *in silico* studies revealed that pilabactam would be anchored to the bottom part of the active site through several electrostatic and also hydrogen-bonding interactions, specifically between (i) the sulfate group and the side chains of residues R250, S118 (SAV-motif), T209 and K208 (KTG-motif); and (ii) the carbonyl group and main NH groups in the catalytic serine residue S70 and residue W211, both involved in the oxyanion hole (Fig. 4A). More importantly, the fluorine atom in pilabactam would establish two key contacts within the residues involved in the tunnel-like entrance of the active site. In particular, the following were observed: (i) a strong hydrogen-bonding interaction with the NH group in residue W157 (Ω-loop) mediated by a water molecule, which is trapped between them during half of the simulation; and (ii) diverse CH–F interactions with the side chains of the residues V120 and L158 (Ω-loop). Both residues W157 and L158 of the Ω-loop play a major role in the catalytic efficiency of the OXA-23 enzyme, as recently revealed by site-directed mutagenesis studies (46). As the fluorine atom is theoretically flanked by the bulky apolar residues V120 and L158 of the tunnel-like entrance, the whole bicyclic system was exquisitely fixed in this pocket throughout the 100-ns simulation (Fig. 3). All of these stabilizing interactions would freeze the position of pilabactam within the catalytic site for serine nucleophilic attack, which arrangement barely fluctuates throughout the simulation, as evidenced by the low RMSD values obtained. A similar binding mode was obtained with the simulation studies performed on the OXA-23/pilabactam covalent adduct, including the CH–F interactions with residues V120 and L158 and the water-mediated interaction with residue W157 (Fig. 4B).

**Fig 5:**
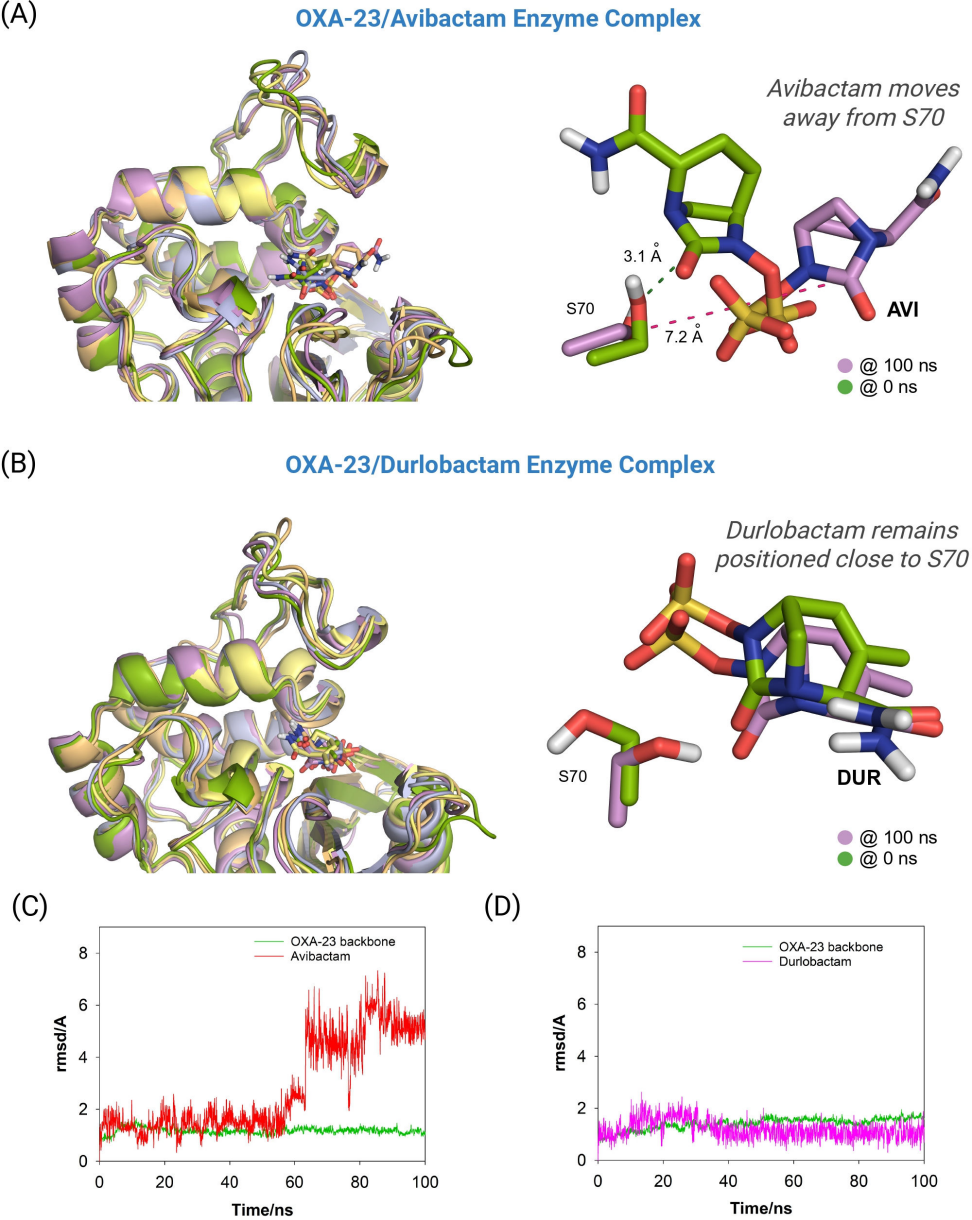
(**A, B**) Superposition of representative snapshots from 100 ns MD simulation of the OXA-23/avibactam (**A**) and OXA-23/durlobactam (**B**) enzyme complexes, highlighting the relative positions of the inhibitors and the catalytic serine residue (S70) at the initial frame and after 100 ns. For avibactam, the reactive centers, the OG atom of S70 and the C2 atom of the ligand’s carbonyl group, became separated during the simulation, whereas durlobactam remained close to S70 throughout. (**C, D**) RMSD plots for the enzyme backbone (Cα, C, O, and N atoms) and inhibitors (avibactam, durlobactam) calculated from 100 ns simulation of the OXA-23/avibactam (**C**) and OXA-23/durlobactam (**D**) enzyme complexes. Average RMSD values for the enzyme backbone were 1.9 and 1.4 Å, respectively, while avibactam and durlobactam exhibited average RMSD values of 2.8 and 1.2 Å, respectively.

Altogether, our simulation studies suggest that replacing the primary carboxamide moiety in avibactam with a fluorine atom, as in pilabactam, enhances interaction of the inhibitor, both in the Michaelis complex and in the covalent adduct, with key residues of the hydrophobic tunnel-like entrance of OXA-23, particularly V120 and L158. These residues constitute a unique structural feature of the enzyme. Additionally, the fluorine substituent promotes indirect interaction with residue W157, a conserved element in OXA-type β-lactamases known to play a critical role in maintaining the correct orientation of the *N*-carboxylated catalytic lysine (K73) during catalysis (Fig. S2) (46). By contrast, simulation studies of the OXA-23/avibactam complex showed that avibactam fails to adopt a stable, geometrically favorable arrangement to support acyl-enzyme adduct formation (Fig. 5A). Specifically, the carbonyl group of avibactam gradually drifts away from the catalytic serine residue during the simulation, ultimately reaching a separation greater than 7 Å, an arrangement incompatible with productive inactivation (Fig. 5A and C).

**Fig 6:**
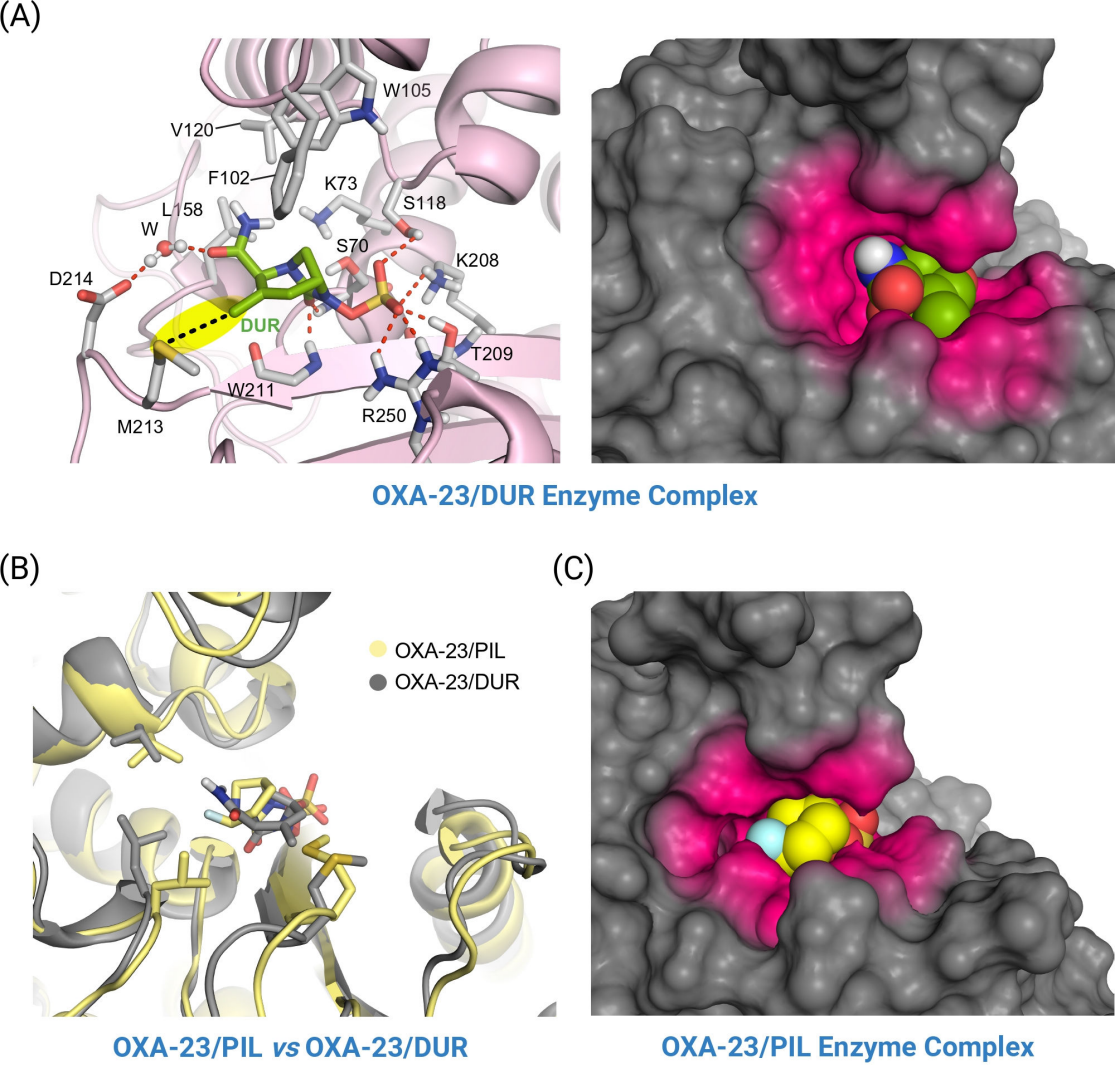
(**A**) Proposed binding mode of durlobactam (green) in the OXA-23 active site (pink) obtained from MD simulation studies. Two representations of the enzyme (cartoon and surface) and the inhibitor (sticks and spheres) are provided. Snapshot taken after 100 ns of dynamic simulation. Relevant side-chain residues (gray) are displayed and labeled. Key hydrogen-bonding interactions are shown by dashed red lines. The C–H····S interaction involving the methyl group in durlobactam is indicated by a yellow shadow and dashed black lines. (**B**) Comparison of OXA-23/pilabactam (yellow) and OXA-23/durlobactam (gray) enzyme complexes. Only the overall enzyme architecture (cartoon), the inhibitors (sticks), and the side-chain residues (sticks) showing different arrangements for both complexes are shown. (**C**) Surface representation of the OXA-23/pilabactam enzyme complex, in which pilabactam is represented as spheres.

For the OXA-23/durlobactam enzyme complex, the results revealed that durlobactam remains highly stable within the catalytic site, adopting a geometry that is well-suited for nucleophilic attack by the catalytic serine residue S70 (Fig. 5B and D). Similar to pilabactam, the ligand is anchored in the active site via its sulfate group, which engages in a network of electrostatic and hydrogen-bonding interactions with residues S118, K208, T209, and R250 (Fig. 6A). Additionally, the carbonyl group forms hydrogen bonds with the backbone NH groups of S70 and W211, key components of the oxyanion hole. The primary carboxamide moiety of durlobactam remains stably positioned throughout the simulation due to a water-mediated hydrogen bond with residue D214. Furthermore, the overall binding is reinforced by a C–H···S interaction (47) between the methyl group and M213, both located on the β5-β6 loop. These favorable interactions collectively result in durlobactam being embedded deep within the active site, facilitating productive acyl-enzyme adduct formation. As expected, superimposition of the binding modes of pilabactam and durlobactam in complex with OXA-23 revealed notable rearrangements in the side chains of residues that interact with their distinct functional groups (i.e., fluorine atom, methyl group, and primary carboxamide) (Fig. 6B). Due to its smaller size and more compact, spherical geometry, pilabactam can penetrate deeper into the catalytic cleft than durlobactam (Fig. 6C). As a result, the closer proximity between the catalytic serine (nucleophile) and the urea moiety (electrophile) in pilabactam may explain the experimentally observed faster formation of the acyl-enzyme adduct.

### Conclusions

In this study, we comprehensively demonstrate that pilabactam effectively restores meropenem activity against CRAB. Using isogenic strains, we show that pilabactam potentiates meropenem against all clinically relevant serine-β-lactamases circulating in *A. baumannii*, including the particularly difficult-to-inhibit CHDLs, thereby reinstating meropenem efficacy by potently inactivating clinically relevant *Acinetobacter*-derived carbapenemases, along with other serine-β-lactamases. The potent activity observed in isogenic strains is corroborated by testing a representative panel of 68 carbapenem-resistant clinical *A. baumannii* isolates collected from Spanish hospitals. These isolates produced CHDLs along with a range of other β-lactam resistance mechanisms. Frequency of resistance studies in four unrelated *A. baumannii* isolates with diverse MEM/PIL MICs reveal a low rate of spontaneous mutations to MEM/PIL at 4× MIC. Concentrations of 16/8 mg/L remain above the *in vitro* mutant selection window and effectively suppress the selection of spontaneous resistant mutants.

Our findings further demonstrate that pilabactam displays strong inhibitory potency against OXA-23 (the most globally prevalent carbapenemase driving resistance in *A. baumannii*), displaying inhibition kinetics comparable to or even better than those of durlobactam. Molecular dynamics simulations, using durlobactam and avibactam as comparators, reveal that the fluorine atom in the pilabactam scaffold plays a critical role in its interaction with the OXA-23 active site. This effect is driven by (i) the small, compact, and spherical geometry of pilabactam; (ii) strong anchoring through multiple electrostatic and hydrogen-bonding interactions with key catalytic residues (S70, S118, K208, W211, R250); and (iii) additional hydrogen-mediated (W157, Ω-loop) and CH–F interactions (V120 and L158, Ω-loop) that influence the tunnel-like entrance of the OXA-23 active site. In combination with meropenem, one of the most effective β-lactams against non-fermenters such as *A. baumannii*, the MEM/PIL pairing emerges as one of the most promising therapeutic candidates currently in development to combat *A. baumannii* infections. These results strongly support the continued progression of this combination through clinical trials.

## MATERIALS AND METHODS

### Construction of an isogenic panel of β-lactamase-producing *A. baumannii* recombinant strains

We engineered an isogenic panel of 15 *A. baumannii* ATCC 17978 strains in order to thoroughly evaluate the inhibitory potency of pilabactam and the spectrum of β-lactamase coverage of the MEM/PIL combination. Each strain expresses a representative β-lactamase-encoding gene from the major Ambler classes circulating in multidrug-resistant *A. baumannii*. The construction of the recombinant strains is detailed in the Supplemental material.

### Whole-genome-sequenced carbapenem-resistant clinical *A. baumannii* strains

All carbapenem-resistant *A. baumannii* clinical isolates were recovered in 2020 as part of the Spanish Nationwide *Acinetobacter* spp. Surveillance Study. We selected 68 *A. baumannii* isolates with meropenem MICs ≥8 mg/L, all previously sequenced by our group (20). Sequencing and bioinformatics methods are described in the Supplemental material.

### Antimicrobial susceptibility testing

MICs were determined for meropenem, MEM/PIL, sulbactam, and SUL/DUR by broth microdilution in cation-adjusted Mueller-Hinton (MH) broth, and also for cefiderocol, which was tested in iron-depleted cation-adjusted MH broth prepared according to CLSI M100 guidelines (48). Further details are provided in the Supplemental material.

### Frequency of resistance and mutant prevention concentrations

The ability of MEM/PIL to suppress the emergence of single-step resistant mutants was assessed at 4×, 8×, and 16× MEM/PIL MICs in four carbapenemase-producing *A. baumannii* clinical isolates (three OXA-23 and one OXA-24/40 producers) from the IHMA global collection. Full experimental procedures are described in the Supplemental material.

### Protein purification

For steady-state kinetic and inhibition studies, the OXA-23 β-lactamase was expressed from pGEX-6P-1 and purified using a glutathione S-transferase (GST) affinity system. Further details are provided in the Supplemental material.

### Steady-state kinetics and inhibition studies

Kinetic characterization was performed using purified enzyme under standardized conditions. Nitrocefin was used as a reporter substrate for enzyme activity, and the steady-state kinetic parameters for hydrolysis were measured using established protocols (19) and are detailed in the Supplemental material. The kinetic parameters of enzymatic inhibition were determined following previously described methodologies (49–52) with further details provided in the Supplemental material.

### Building of the Michaelis complexes by molecular docking

Molecular docking studies were performed using the GOLD software package (version 2022.3.0) (39). The enzyme coordinates were obtained from the crystal structure of *A. baumannii* OXA-23 covalently bound to meropenem (PDB ID 4JF4 [40]; 2.14 Å resolution). The geometries of pilabactam, durlobactam, and avibactam were minimized using the AM1 Hamiltonian implemented in Gaussian 09 (53) and saved as MOL2 files for docking. Ligands were docked using 25 independent genetic algorithm (GA) runs, each consisting of up to 100,000 GA operations on a single population of 50 individuals. Default operator weights were used for crossover, mutation, and migration (95, 95, and 10, respectively). Hydrogen bonding and van der Waals distance cut-offs were set at 4.0 and 2.5 Å, respectively. The catalytic serine residue defined the center of the docking region, with a spherical search space of 6 Å radius. All crystallographic water molecules and ions were removed prior to docking. The “flip ring corners” option was enabled, while default settings were used for all other flags. The GOLD scoring function was used to evaluate docking poses.

### Molecular dynamics simulation studies on the OXA-23/ligand enzyme complexes and adducts

The proposed binding modes obtained from molecular docking were embedded in a truncated octahedral box of TIP3P water molecules and neutralized by the addition of sodium ions. MD simulations were conducted using the ff14SB (41) force field in AMBER 21. Ligand minimization, construction and minimization of the OXA-23/inhibitor adducts and binary complexes, as well as the subsequent 100 ns MD simulations, were performed according to our previously described protocol (42, 43). The stability and reliability of the resulting OXA-23/ligand complexes and covalent adducts were assessed by calculating the RMSD of the protein backbone atoms (Cα, C, N, and O), the ligands, and the covalently modified residues throughout the simulation using the cpptraj module of AMBER 21. Molecular visualizations and structural depictions were generated using PyMOL (54). The SAND consensus numbering for class D enzymes was used (55). Figure 3 to 6 were created using the BioRender program (https://www.biorender.com/).

## SUPPLEMENTAL MATERIAL

Supplemental material aac.01691-25-s0001.docx Tables S1 and S2; Fig. S1 and S2.

ASM does not own the copyrights to Supplemental Material that may be linked to, or accessed through, an article. The authors have granted ASM a non-exclusive, world-wide license to publish the Supplemental Material files. Please contact the corresponding author directly for reuse.
