# Supplementary material for: Disarming carbapenemase-producing Acinetobacter baumannii: high potency of the novel therapeutic combination of meropenem and the innovative diazabicyclooctane β-lactamase inhibitor pilabactam (formerly ANT3310)
Source: Antimicrob Agents Chemother. 2026 Feb 19;70(4):e01691-25. doi: 10.1128/aac.01691-25 (PMC13041389; doi:10.1128/aac.01691-25)
Supplement: Supplemental material — Tables S1 and S2; Fig. S1 and S2. [file aac.01691-25-s0001.docx]

**SUPPLEMENTARY MATERIAL**

**Disarming Carbapenemase-Producing *Acinetobacter baumannii*: High Potency of the Novel Therapeutic Combination of Meropenem and the Innovative Diazabicyclooctane β-Lactamase Inhibitor Pilabactam (formerly ANT3310)**

Salud Rodríguez-Pallares^1,2^*, Michelle Outeda-García^1^*, Emilio Lence^3,4^, Arianna Rodríguez-Coello^1^, Lucía González-Pinto^1,2^, Gabriela Alejandra Báez-Barroso^1^, Tania Blanco-Martín^1,2^, Juan Carlos Vázquez-Ucha^1,2^, Agustina Llanos^5^, Filomena Sannio^6^, Jean-Denis Docquier^6^, Ian Morrissey^7^, Stephen Howser^8^, Magdalena Zalacain^5^, Marc Lemonnier^5^, Concepción González-Bello^3^, Germán Bou^1,2,9^, Alejandro Beceiro^1,2^** and Jorge Arca-Suárez^1,2^**

1. Servicio de Microbiología Clínica & Grupo de Investigación en Microbiología. Instituto de Investigación Biomédica de A Coruña (INIBIC), Complexo Hospitalario Universitario de A Coruña (CHUAC), Sergas. Universidade da Coruña (UDC), A Coruña, Spain
2. CIBER de Enfermedades Infecciosas (CIBERINFEC), Instituto de Salud Carlos III, Madrid, España
3. Centro Singular de Investigación en Química Biolóxica e Materiais Moleculares (CiQUS), Departamento de Química Orgánica, Universidade de Santiago de Compostela, Santiago de Compostela, Spain.
4. Departamento de Química Orgánica, Facultad de Ciencias, Universidad de Valladolid, Campus Miguel Delibes, Valladolid, Spain
5. Antabio SAS, Labège, France
6. Dipartimento di Biotecnologie Mediche, Università degli Studi di Siena, Siena, Italy
7. Antimicrobial Focus Ltd., Sawbridgeworth, UK
8. IHMA Europe, Monthey (Valais), Switzerland
9. Department of Physiotherapy, Medicine and Biomedical Sciences, University of A Coruña, A Coruña, Spain

*Salud Rodríguez-Pallares and Michelle Outeda-García contributed equally to this work and share first autorship.

**Alejandro Beceiro and Jorge Arca-Suárez contributed equally to this work and share last authorship.

**Corresponding author:** Dr. Jorge Arca-Suárez

e-mail: jorge.arca.suarez@sergas.es

Servicio de Microbiología Clínica & Grupo de Investigación en Microbiología. Instituto de Investigación Biomédica de A Coruña (INIBIC), Complexo Hospitalario Universitario de A Coruña (CHUAC), Sergas. Universidade da Coruña (UDC), As Xubias S/N, 3º Planta, 15006, A Coruña, Spain

CIBER de Enfermedades Infecciosas (CIBERINFEC), Instituto de Salud Carlos III, Madrid, España

Phone: 0034679082502

**Construction of an isogenic panel of β-lactamase-producing *A. baumannii* recombinant strains**

The following representative Ambler class A, B, and D β-lactamase genes were cloned into *A. baumannii* ATCC 17978: class A (*bla*_GES-1_, *bla*_GES-5_, *bla*_CTX-M-15_, *bla*_SHV-12_, *bla*_PER-1_, *bla*_TEM-52_, *bla*_KPC-3_), class B (*bla*_IMP-2_, *bla*_NDM-1_) and class D (*bla*_OXA-23_, *bla*_OXA-24/40_, *bla*_OXA-51-like_, *bla*_OXA-58_, *bla*_OXA-143_, *bla*_OXA-235_). The recombinant strains producing class D β-lactamases were previously constructed by our research group (1). For the remaining enzymes, each β-lactamase gene was amplified from whole-genome sequenced clinical isolates available in our laboratory, using PrimeSTAR HS DNA polymerase (Takara, Berkeley, CA). PCR products were ligated into the pET-RA + KmR plasmid (containing a kanamycin resistance marker) under control of the *bla*_CTX-M-14_ promoter previously cloned into this vector (2). The resulting constructs were initially transformed into *Escherichia coli* TG1 reference strain. Recombinant plasmids were then electroporated into wild-type *A. baumannii* ATCC 17978. Final transformants were selected on Luria Bertani (LB) agar plates supplemented with 50 mg/L kanamycin. All constructs were validated by PCR, Sanger sequencing, plasmid restriction analysis and phenotypic testing.

**Whole-genome-sequenced (WGS) carbapenem-resistant clinical *A. baumannii* strains**

The Spanish Nationwide *Acinetobacter* spp. Surveillance Study conducted by our group included isolates collected from 24 participating hospitals (3). The 68 selected strains had previously been sequenced using short-read (Illumina) and long-read (Oxford Nanopore Technologies) platforms, in combination or separately, enabling in-depth bioinformatic analysis to define β-lactam resistance mechanisms. These included the acquisition of β-lactamase genes via horizontal gene transfer, the presence of insertion sequences (IS) associated with β-lactamase overexpression, alterations in PBPs, and loss-of-function mutations affecting either negative regulators of efflux pump operons or genes encoding outer membrane porins.

**Antimicrobial susceptibility testing**

MICs were determined by broth microdilution according to CLSI M100 (4). Pilabactam was tested at a fixed concentration of 8 mg/L. Durlobactam was tested, at a fixed concentration of 4 mg/L, in combination with sulbactam against both clinical and isogenic isolates. It was also evaluated at fixed concentrations of 4 and 8 mg/L in combination with meropenem against isogenic transformants, to enable direct comparison with pilabactam. CLSI M100 breakpoints were applied for meropenem, cefiderocol, sulbactam and sulbactam/durlobactam (SUL/DUR) (4). For the meroepenem/pilabactam (MEM/PIL) combination, a susceptibility breakpoint of 8 mg/L was used, based on pharmacokinetic/pharmacodynamic (PK/PD) data supporting a dosing regimen of 2 g administered intravenously over 3 hours every 8 hours (5). This regimen is considered safe and is well tolerated and achieves >90% probability of target attainment (PTA) for isolates with MICs ≤8 mg/L, and it is currently being used in ongoing clinical trials of the MEM/PIL combination (6). Quality control was performed using *E. coli* ATCC 25922 and *Klebsiella pneumoniae* ATCC BAA-2814 as reference strains.

**Frequency of Resistance (FoR) and Mutant Prevention Concentrations (MPC)**

The agar MICs were determined for each isolate following CLSI recommendations and were confirmed under the frequency of resistance study conditions in 14 cm agar plates. The MIC was defined as the lowest antibiotic concentration inhibiting formation of a bacterial lawn and/or patch of colonies. For each parental strain, approximately 2 × 10^9^ cells from fresh Mueller–Hinton (MH) broth cultures were plated onto 20 MH agar plates with 4×, 8× or 16× the respective baseline MEM/PIL agar MICs. Serial dilutions of the starting inocula were spread onto TSA plates to determine the exact amount of inoculum. Plates were incubated at 37 °C for 24 hours. All colonies isolated under these conditions were sub-cultured five times on drug-free agar plates, and MICs were determined by broth microdilution following CLSI susceptibility testing standards. Isolates were considered resistant mutants if the MIC was >2-times higher than the MIC of the parent strain. The frequency of spontaneous mutation was determined as the proportion of confirmed resistant mutants in the total population for each concentration of MEM/PIL tested. If no mutants were observed, the mutation frequency was defined as less than the mutation frequency calculated if one mutant had been observed. The mutant prevention concentration (MPC) was then defined as the lowest concentration at which no spontaneous mutants were detected.

**Protein purification**

To purify the OXA-23 enzyme, the *bla*_OXA-23_ gene, excluding the sequence encoding the signal peptide, was amplified and cloned into the pGEX-6P-1 vector using *Bam*HI and *Eco*RI restriction sites. This construct produced a fusion protein comprising glutathione S-transferase (GST) and the target enzyme. Cloning procedures followed the protocol described above, and primers 5’-CGCGGATCCTTAATAAATGAAACCCCGAGTC-3’ and 5’-CCGGAATTCTTAAATAATATTCAGCTGTTTTAATG-3’ were used. The recombinant plasmids were introduced into *E. coli* BL21 via electroporation, and transformants were selected on LB agar plates supplemented with 100 mg/L ampicillin. The recombinant β-lactamase was purified to homogeneity using the GST fusion system (Cytiva, Marlborough, Massachusetts, USA) according to the manufacturer’s instructions. Purity was confirmed by SDS-PAGE, revealing a single band of approximately 29 kDa, corresponding to ≥99% purity. The OXA-24 protein was purified in a similar manner following previously published procedures (7, 8).

**Steady-state kinetics and inhibition studies**

Kinetic characterization was performed using purified enzyme under standardized conditions. Nitrocefin served as the reporter substrate with measurements performed at 490 nm (ε = 15,900 M⁻¹ cm⁻¹). Purified enzyme was mixed with various concentrations of nitrocefin in phosphate-bicarbonate buffer (50 mM sodium phosphate and 20 mM sodium bicarbonate, pH 7.2). Substrate hydrolysis was monitored spectrophotometrically using an Epoch 2 microplate reader (BioTek, VT, USA) at 25°C under steady-state conditions, as previously described (1), with all determinations performed in triplicate. The Michaelis constant (*K_m_*) and turnover number (*k_cat_*) for nitrocefin were determined following established β-lactamase protocols (9). The resulting steady-state parameters were *K_m_*= 135 µM and *k_cat_*= 101 s⁻¹, resulting in a catalytic efficiency (*k_cat_*/*K_m_*) of 0.75 µM⁻¹ s⁻¹.

To determine the apparent inhibition constant (*K*_i app_) for enzyme-inhibitor binding affinity, the purified enzyme was incubated in the presence of variable concentrations of the inhibitor and a fixed concentration of nitrocefin (the reporter substrate) in reaction buffer, whose rate of hydrolysis (*V*₀) were measured spectrophotometrically, according to established protocols (10). The resulting data were analysed using the Dixon transformation (1/*V*₀ *vs.* inhibitor concentration [I]), and *K*ᵢ _app_ values were calculated from the intercept-to-slope ratio. Initial velocities (V₀) were determined using Equation 1 (11):

1 - *V*_0_= (*V*_max_ × [S] / (*K*_mNFC_ x (1+ ([I]/*K*_i_)) + [S])

2- *K*_i app_ (corrected)= *K*_i app_ (observed) / [ 1+ ([S]/*K*_mNFC_)]

The inactivation process follows a two-step mechanism in which the enzyme (E) and inhibitor (I) first form a reversible non-covalent complex (E·I) with equilibrium constant *K*, followed by a acylation step (k₊₂) leading to the covalent adduct (E–I). The overall inactivation rate, *K_inact_*, depends on the inhibitor concentration [I]; When *K>>* [I], the expression simplifies to a linear dependence where only the ratio *k_+2_/K* (the apparent second-order rate constant) can be determined experimentally.


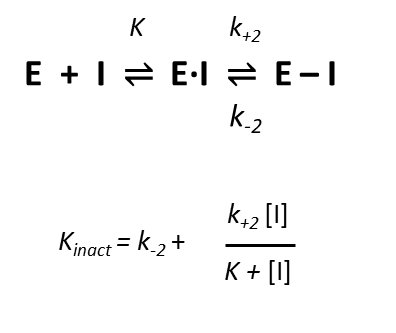


*k*_inact_/*K*_I_ and *K*_+2_/*K* are the second-order rate constants that are generally used for comparing inhibitor efficiencies. The inhibitor-mediated enzyme inactivation rate (*k*_inact_) was measured in the presence of nitrocefin and the *K*_I_ determined as previously described (1, 12). The *k*_obs_ values were determined using nonlinear least-squares fit of the data, employing GraphPad Prism software (Dotmatics, Boston, USA), following previously established protocols (10). When the *k*_obs_ *vs.* [I] graph yielded a hyperbola, the data were fitted to equation 3 to obtain the values of *k_inact_* and *K*_I_ that represent the maximum rate of inactivation and the inhibitor concentration that yields half the maximum rate of inactivation, respectively. The *K*_I_ value was corrected for nitrocefin affinity using the same correction model as in Equation 2, adapted for *K*_I_.

When *K_app_* is much greater than the highest tested inhibitor concentration, then Equation 3 simplifies to *k*_obs_ = ((*k*_+2_ ​[I])/​*K*_app_​), yielding a linear *k*_obs_ *vs*. [I] graph, whose slope is *k*_+2_/*K_app_*. The *K_app_* value was also corrected for nitrocefin affinity an analogous correction model as in Equation 2, to yield *K* (12, 13).

3- *k*_obs_ ​= *k*_-2_ ​+ ((*k*_+2_ ​[I])/​*K*_app_​)

Dissociation rate constants (*k*_off_) were determined using rapid dilution methodology to measure enzyme-inhibitor complex dissociation kinetics. Purified OXA-23 enzyme (1 μM) was mixed with inhibitor at a concentration 10x *K*ᵢ _app_ for 30 minutes at 37°C to ensure complete enzyme inactivation. The reaction mixture was then diluted 10,000-fold in reaction buffer, and 197 μL of diluted enzyme was combined with 3 μL of nitrocefin (3× *K*_m_ concentration) in the reaction buffer.

Enzyme activity recovery was monitored spectrophotometrically at 490 nm every minute for 4 hours at room temperature. The resulting reactivation profiles were fitted Equation 4 (12, 14):

4- P = *V*_s_ x *t* + (*V*_0_-*V*_s_) x [1– exp ^(-^*^k^*^off x^ *^t^*^)^] / *k*_off_

where P is the product concentration (i.e. hydrolyzed nitrocefin, determined using the absorbance measured at 490 nm); *V*_s_ is the steady-state uninhibited enzyme velocity and *V*₀ is the initial completely inhibited enzyme velocity. Furthermore, the dissociation half-life (*t*_1/2_) was computed by GraphPad Prism software (Dotmatics, Boston, USA) during the curve-fitting process.

Finally, the equilibrium dissociation constant (*K*_d_) was calculated as *K*_d_ = *k*_off_/*k*_on_ from the experimentally determined rate constants (with *k*_on_ being *on-rate* constant for the formation of the enzyme-inhibitor complex, which we assume to be comparable to *k_+_*_2_/*K*).

Supplementary Table 1**.** Sequence types, β-lactamases and susceptibility to β-lactams and β-lactam/β-lactamase inhibitor combinations against carbapenem-resistant *A. baumannii* clinical isolates.

| **Isolate ID** | **Hospital** | **ST** | **MIC (mg/L)** | | | | | **β-lactamases** | **Porins and PBPs** |
| --- | --- | --- | --- | --- | --- | --- | --- | --- | --- |
|  |  |  | **MEM (R≥8) ^a^** | **M/P (R≥8) ^b^** | **SUL**  **(R≥16) ^c^** | **S/D (R≥16) ^a^** | **FDC (R≥16) ^a^** |  |  |
| 4 | HOS1 | 2 | 32 | 2 | 8 | 2 | ≤0.125 | ADC-30, OXA-66, OXA-23 |  |
| 5 | HOS1 | 2 | 32 | 1 | 8 | 2 | ≤0.125 | ADC-30, OXA-66, -OXA-23 |  |
| 6 | HOS1 | 2 | 32 | 1 | 8 | 2 | ≤0.125 | ADC-30, OXA-66, OXA-23, TEM-12 |  |
| 7 | HOS1 | 2 | 32 | 1 | 16 | 4 | 2 | ADC-30, OXA-66, OXA-23 |  |
| 8 | HOS1 | 2 | 32 | 1 | 8 | 2 | ≤0.125 | ADC-30, OXA-66, OXA-23 |  |
| 9 | HOS2 | 2 | 32 | 2 | 16 | 2 | 0.25 | ADC-30, OXA-66, OXA-23, TEM-12 |  |
| 10 | HOS1 | 2 | 32 | 1 | 8 | 2 | 0.5 | ADC-30, OXA-66, OXA-23 |  |
| 12 | HOS3 | 2 | 16 | 2 | 2 | 2 | 0.5 | ADC-30, IS*Aba*1-OXA-201 |  |
| 13 | HOS3 | 2 | 16 | 1 | 2 | 2 | 0.25 | ADC-30, IS*Aba*1-OXA-201 |  |
| 14 | HOS3 | 2 | 16 | 2 | 2 | 2 | 0.25 | ADC-30, IS*Aba*1-OXA-201 |  |
| 15 | HOS3 | 2 | 16 | 1 | 2 | 2 | ≤0.125 | ADC-30, IS*Aba*1-OXA-201 |  |
| 16 | HOS3 | 2 | 16 | 1 | 4 | 2 | ≤0.125 | ADC-30, IS*Aba*1-OXA-201 |  |
| 17 | HOS3 | 2 | 32 | 0.5 | 4 | 2 | 0.25 | ADC-30, IS*Aba*1-OXA-201 |  |
| 18 | HOS3 | 2 | 16 | 0.5 | 2 | 2 | ≤0.125 | ADC-30, IS*Aba*1-OXA-201 |  |
| 19 | HOS3 | 2 | 16 | 1 | 8 | 1 | ≤0.125 | ADC-30, OXA-66, OXA-58 |  |
| 20 | HOS3 | 2 | 16 | ≤0.125 | 2 | 1 | 1 | ADC-30, IS*Aba*1-OXA-201 |  |
| 24 | HOS3 | 2 | 16 | 2 | 2 | 2 | 0.25 | ADC-30, IS*Aba*1-OXA-201 |  |
| 25 | HOS3 | 2 | 16 | 2 | 2 | 2 | ≤0.125 | ADC-30, IS*Aba*1-OXA-201 |  |
| 26 | HOS3 | 2 | 8 | 2 | 4 | 1 | ≤0.125 | ADC-30, OXA-66, OXA-58 |  |
| 28 | HOS4 | 2 | 32 | 1 | 16 | 2 | 0.5 | ISAba1-ADC-30, OXA-66, OXA-23 |  |
| 29 | HOS4 | 2 | 32 | 1 | 8 | 2 | ≤0.125 | ADC-30, OXA-66, OXA-23 |  |
| 30 | HOS4 | 2 | 32 | 1 | 8 | 2 | 0.25 | ISAba1-ADC-30, OXA-66, OXA-23 |  |
| 31 | HOS4 | 2 | 32 | 1 | 16 | 1 | ≤0.125 | ADC-30, OXA-66, OXA-23 |  |
| 32 | HOS4 | 2 | 32 | 1 | 4 | 1 | 0.25 | ISAba1-ADC-30, OXA-66, OXA-23 |  |
| 33 | HOS4 | 2 | 32 | 2 | 8 | 2 | ≤0.125 | ADC-30, OXA-66, OXA-23 |  |
| 35 | HOS4 | 2 | 64 | 1 | 8 | 2 | 0.25 | ADC-30, OXA-66, OXA-23 |  |
| 36 | HOS4 | 2 | 32 | 2 | 8 | 2 | ≤0.125 | ADC-30, OXA-66, OXA-23 |  |
| 37 | HOS4 | 2 | 32 | 1 | 8 | 1 | 0.25 | ADC-30, OXA-66, OXA-23 |  |
| 39 | HOS4 | 2 | 8 | 0.25 | 4 | 1 | ≤0.125 | ADC-30, OXA-66, OXA-23 |  |
| 41 | HOS4 | 2 | 32 | 1 | 8 | 2 | 0.25 | ADC-30, OXA-66, OXA-23 |  |
| 42 | HOS5 | 2 | 32 | 1 | 8 | 1 | ≤0.125 | ADC-30, OXA-66, OXA-23 |  |
| 44 | HOS6 | 2 | 32 | ≤0.125 | 4 | 0.25 | ≤0.125 | ADC-30, OXA-66, OXA-23 |  |
| 45 | HOS6 | 1 | 64 | 1 | 8 | 0.5 | ≤0.125 | ADC-75, OXA-69, OXA-23 |  |
| 46 | HOS6 | 1 | 64 | 1 | 8 | 0.5 | ≤0.125 | ADC-75, OXA-69, OXA-23 |  |
| 47 | HOS6 | 1 | 64 | 1 | 8 | 0.5 | ≤0.125 | ADC-75, OXA-69, OXA-23 | Omp33-36 ΔV43insT& A45insQ |
| 49 | HOS6 | 1 | 64 | 2 | 16 | 1 | ≤0.125 | ADC-75, OXA-69, OXA-23 | Omp33-36 ΔV44insT& A45insQ |
| 52 | HOS6 | 1 | 64 | 2 | 8 | 0.5 | ≤0.125 | ADC-75, OXA-69, OXA-23 |  |
| 53 | HOS6 | 1 | 64 | 0.5 | 8 | 0.5 | ≤0.125 | ISAba1-ADC-75, OXA-69, OXA-23 | Omp33-36 A45insQ Omp25 V234fs PBP3 E169fs |
| 54 | HOS6 | 1 | 64 | 1 | 8 | 0.5 | ≤0.125 | ADC-75, OXA-69, OXA-23 |  |
| 55 | HOS6 | 1 | 64 | 1 | 8 | 0.5 | ≤0.125 | ADC-75, OXA-69, OXA-23 |  |
| 56 | HOS6 | 1 | 64 | 0.25 | 16 | 0.25 | ≤0.125 | ADC-75, OXA-69, OXA-23 | OprD Q315fs |
| 83 | HOS7 | 25 | 64 | 0.5 | 8 | 0.25 | ≤0.125 | ADC-5, OXA-64, OXA-23 |  |
| 109 | HOS8 | 1 | 64 | 0.5 | 8 | 0.5 | ≤0.125 | ADC-75, OXA-69, OXA-23 |  |
| 133 | HOS9 | 2 | 8 | 1 | 16 | 0.5 | ≤0.125 | ADC-30, OXA-66, OXA-58, TEM-12 |  |
| 134 | HOS9 | 2 | 8 | 0.25 | 16 | ≤0.125 | ≤0.125 | ADC-30, OXA-66, OXA-58, TEM-12 |  |
| 137 | HOS10 | 85 | 16 | 1 | 8 | 2 | ≤0.125 | ADC-2, OXA-94, OXA-23 |  |
| 138 | HOS10 | 745 | 16 | 0.5 | 16 | 0.25 | 0.5 | ADC-30, OXA-66, OXA-58 |  |
| 140 | HOS10 | 85 | 64 | 2 | 32 | 4 | 0.25 | ADC-2, OXA-94, OXA-23 |  |
| 141 | HOS10 | 745 | 16 | 0.5 | 8 | 0.25 | ≤0.125 | ADC-30, OXA-66, OXA-58 |  |
| 144 | HOS10 | 745 | 16 | 1 | 4 | 0.25 | ≤0.125 | ADC-30, OXA-66, OXA-58 |  |
| 148 | HOS10 | 745 | 8 | 0.5 | 8 | 1 | 0.25 | ADC-30, OXA-66, OXA-58 |  |
| 169 | HOS11 | 2 | 32 | 2 | 8 | 1 | 0.25 | ADC-30, OXA-66, OXA-23 |  |
| 171 | HOS11 | 2 | 32 | 2 | 16 | 4 | ≤0.125 | ADC-30, OXA-66, OXA-23 |  |
| 173 | HOS11 | 2 | 32 | 1 | 32 | 1 | 2 | IS*Aba*1-ADC-30, OXA-66, OXA-23 |  |
| 174 | HOS11 | 2 | 64 | 1 | 8 | 0.5 | ≤0.125 | ADC-30, OXA-66, OXA-23 |  |
| 179 | HOS12 | 2 | 64 | 2 | 8 | 1 | ≤0.125 | ADC-30, OXA-66, OXA-23 |  |
| 180 | HOS12 | 2 | >64 | ≤0.125 | 16 | 1 | ≤0.125 | ADC-30, OXA-66, OXA-24/40 |  |
| 181 | HOS12 | 2 | 64 | 2 | 8 | 1 | ≤0.125 | ADC-30, OXA-66, OXA-23 |  |
| 182 | HOS12 | 2 | 16 | 0.5 | 16 | 2 | ≤0.125 | ADC-30, OXA-66, OXA-23 |  |
| 183 | HOS12 | 2 | 32 | ≤0.125 | 8 | ≤0.125 | ≤0.125 | ADC-30, OXA-66, OXA-23 |  |
| 184 | HOS12 | 2 | 32 | 1 | 32 | 1 | 1 | ADC-30, OXA-66, OXA-23 |  |
| 186 | HOS12 | 2 | 64 | 0.5 | 4 | 0.5 | ≤0.125 | ADC-30, OXA-66, OXA-23 |  |
| 191 | HOS12 | 2 | 64 | 0.5 | 8 | 1 | ≤0.125 | ADC-30, OXA-66, OXA-23 |  |
| 192 | HOS12 | 2 | 32 | 0.5 | 8 | 1 | ≤0.125 | ADC-30, OXA-66, OXA-23 |  |
| 194 | HOS12 | 2 | 32 | 1 | 8 | 2 | ≤0.125 | ADC-30, OXA-66, OXA-23 |  |
| 199 | HOS12 | 2 | 16 | 0.5 | 8 | 2 | 0.5 | ADC-30, OXA-66, OXA-23 |  |
| 201 | HOS12 | 2 | 64 | 1 | 4 | 1 | ≤0.125 | ADC-30, OXA-66, OXA-23 | OprD Q33* |
| 204 | HOS12 | 2 | 32 | 0.5 | 16 | 0.5 | ≤0.125 | ADC-30, OXA-66, OXA-23 | CarO W57* |
| ST, sequence type; MEM, meropenem; M/P, meropenem/pilabactam; SUL, sulbactam; S/D, sulbactam/durlobactam; FDC, cefiderocol; *, stop codon  ^a^ 2025 CLSI breakpoint indicated  ^b^ For M/P a breakpoint of 8 mg/L was applied  ^c^ For SUL a breakpoint of 16 mg/L was applied | | | | | | | | | |

Supplementary Table 2. Summary of the frequency of spontaneous mutants and mutant prevention concentration (MPC) values of 4 representative carbapenem-resistant, CHDL-producing *A. baumannii* isolates.

| **Strain #** | **Species and CHDLs** | **M/P Agar MIC (mg/L)** | **FoR**  **condition** | **FoR**  **results** | **MPC (mg/L)** |
| --- | --- | --- | --- | --- | --- |
| NTBC094 | *A. baumannii*  **OXA-23** | 2 | 4× MIC | < 5.3x10^-10^ | 8 |
|  |  |  | 8× MIC | < 5.3x10^-10^ |  |
|  |  |  | 16× MIC | < 5.3x10^-10^ |  |
| 1631176 | *A. baumannii*  **OXA- 23** | 4 | 4× MIC | < 1.2x10^-9^ | 16 |
|  |  |  | 8× MIC | < 1.2x10^-9^ |  |
|  |  |  | 16× MIC | < 1.2x10^-9^ |  |
| 1631947 | *A. baumannii*  **OXA- 23** | 1 | 4× MIC | < 2.8x10^-10^ | 4 |
|  |  |  | 8× MIC | < 2.8x10^-10^ |  |
|  |  |  | 16× MIC | < 2.8x10^-10^ |  |
| 1630177 | *A. baumannii*  **OXA- 24** | 1 | 4× MIC | < 4.2x10^-10^ | 4 |
|  |  |  | 8× MIC | < 4.2x10^-10^ |  |
|  |  |  | 16× MIC | < 4.2x10^-10^ |  |
| M/P, meropenem/pilabactam; FoR, frequency of resistance | | | | |  |


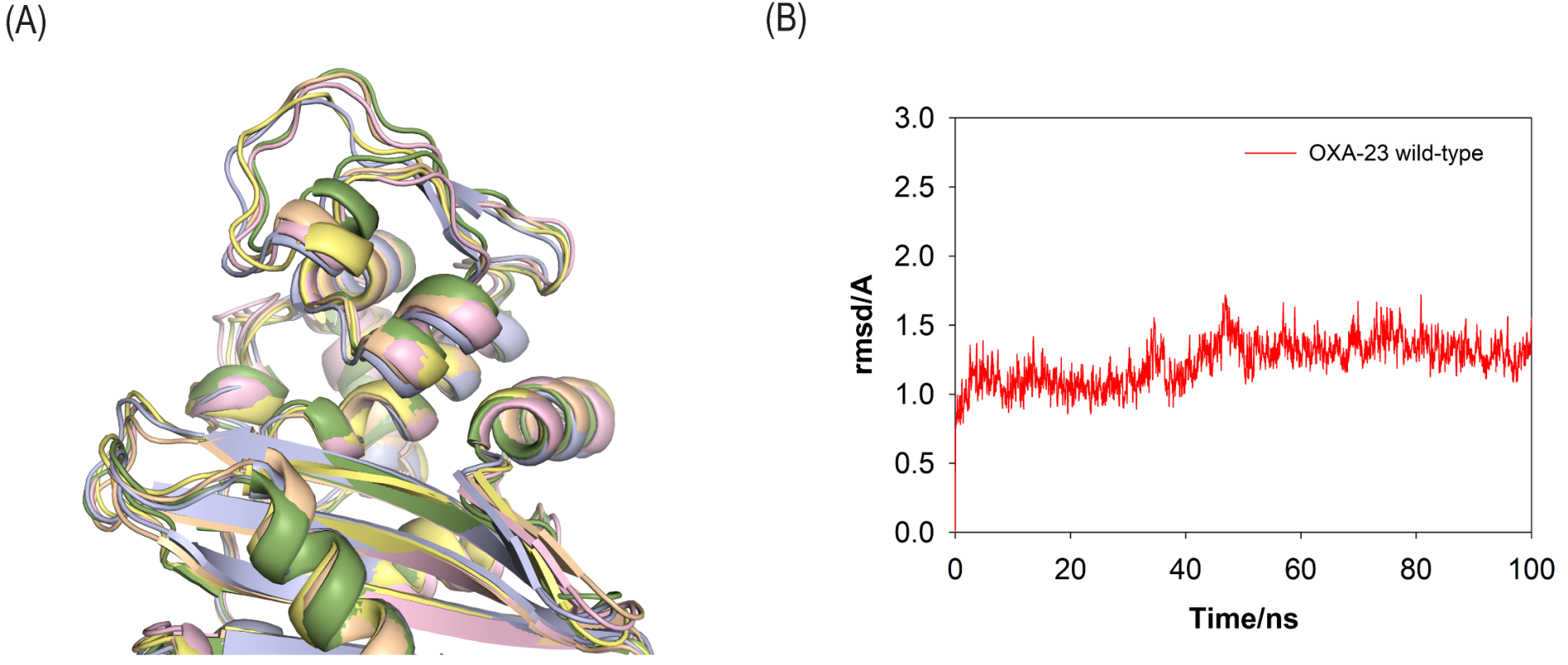


Supplementary Figure 1. (A) Superposition of several snapshots from the 100 ns of MD simulation on the OXA-23 enzyme in the free form. (B) Root-mean-square deviations (RMSD) plot for the protein backbone (Cα, C, O and N atoms) calculated from the 100 ns MD simulation of OXA-23 enzyme in the free form. An average low RMSD value of 1.2 Å was obtained. Note how no significant changes are identified during simulation.


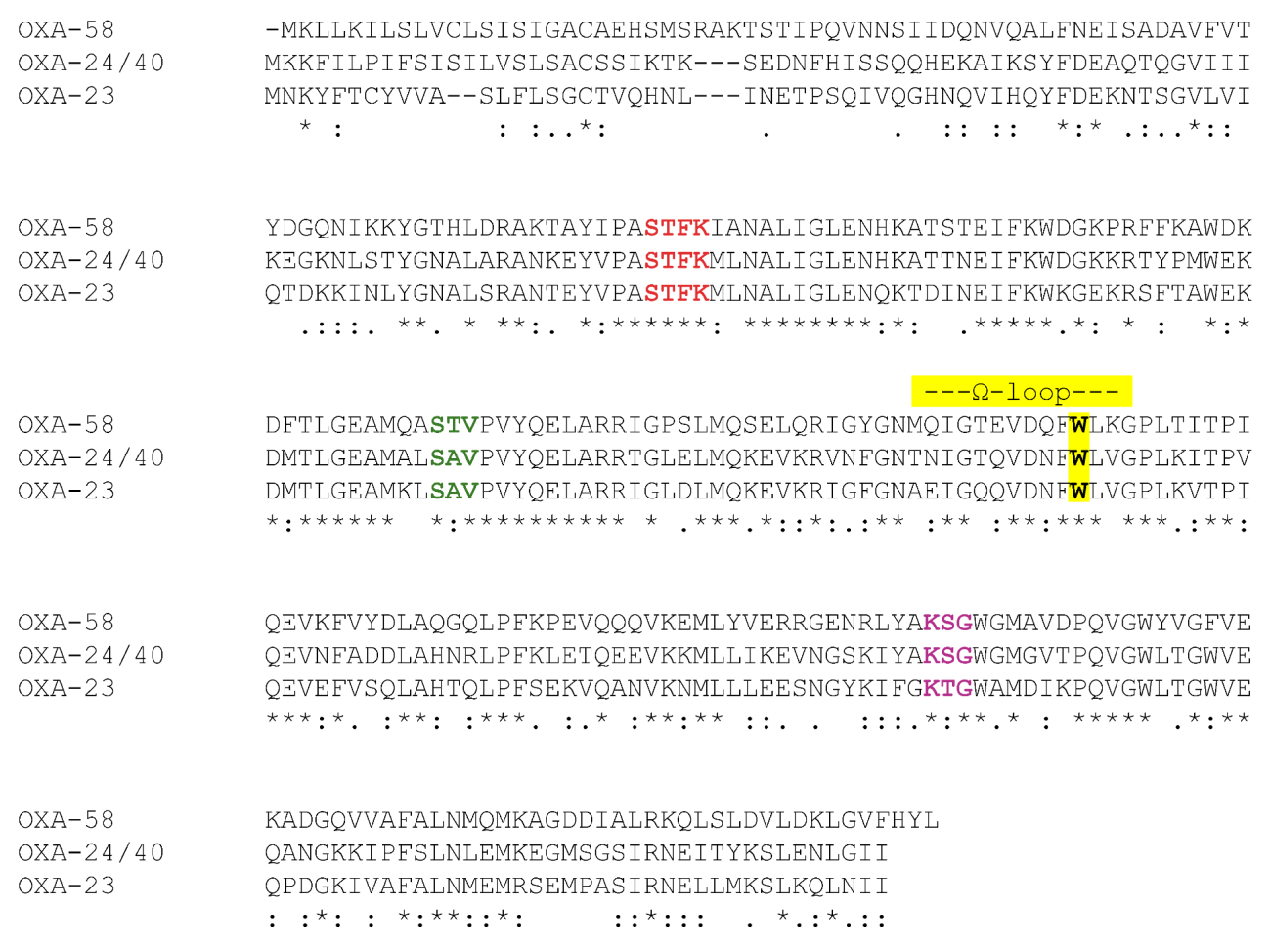


Supplementary Figure 2. Multiple sequence alignment. Amino acid sequence alignments for OXA-58, OXA-24/40, and OXA-23 enzymes. Protein sequences were aligned using the CLUSTAL Omega (1.2.4) multiple sequence alignment (<https://www.ebi.ac.uk/jdispatcher/msa/clustalo>, accessed on September 24, 2025). Fully conserved (*) and functionally conserved (:) residues are highlighted. The three conserved motifs (STFK, SAV and KTG) in OXA enzymes are displayed in red, green and purple, respectively. The location of the Ω-loop and the conserved residue W157 are indicated in yellow.

**References**

1. Vázquez-Ucha JC, Maneiro M, Martínez-Guitián M, Buynak J, Bethel CR, Bonomo RA, Bou G, Poza M, González-Bello C, Beceiro A. 2017. Activity of the ß-Lactamase inhibitor LN-1-255 against carbapenem-hydrolyzing class D ß-Lactamases from *Acinetobacter baumannii*. Antimicrob Agents Chemother 61:e01172-17.

2. Fernández A, Pérez A, Ayala JA, Mallo S, Rumbo-Feal S, Tomás M, Poza M, Bou G. 2012. Expression of OXA-type and SFO-1 β-lactamases induces changes in peptidoglycan composition and affects bacterial fitness. Antimicrob Agents Chemother 56:1877–1884.

3. Lasarte-Monterrubio C, Guijarro-Sánchez P, Alonso-Garcia I, Outeda M, Maceiras R, González-Pinto L, Martínez-Guitián M, Fernández-Lozano C, Vázquez-Ucha JC, Bou G, Arca-Suárez J, Beceiro A. 2024. Epidemiology, resistance genomics and susceptibility of *Acinetobacter* species: results from the 2020 Spanish nationwide surveillance study. Euro Surveill 29:2300352.

4. Clinical and Laboratory Standards Institute. 2024. Performance standards for antimicrobial susceptibility testing, 34th ed. CLSI supplement M100. Clinical and Laboratory Standards Institute, Wayne, PA.

5. Novelli A, Del Giacomo P, Rossolini GM, Tumbarello M. 2020. Meropenem/vaborbactam: a next generation β-lactam β-lactamase inhibitor combination. Expert Rev Anti Infect Ther 18:643–655.

6. Lee NY, Tsai CS, Syue LS, Chen PL, Li CW, Li MC, Ko WC. 2020. Treatment Outcome of Bacteremia Due to Non–Carbapenemase-producing Carbapenem-Resistant *Klebsiella pneumoniae* Bacteremia: Role of Carbapenem Combination Therapy. Clin Ther 42:e33–e44.

7. Santillana E, Beceiro A, Bou G, Romero A. 2007. Crystal structure of the carbapenemase OXA-24 reveals insights into the mechanism of carbapenem hydrolysis. Proc Natl Acad Sci U S A 104:5354-9.

8. Lahiri SD, Mangani S, Jahić H, Benvenuti M, Durand-Reville TF, De Luca F, Ehmann DE, Rossolini GM, Alm RA, Docquier JD. 2015. Molecular basis of selective inhibition and slow reversibility of avibactam against class D carbapenemases: a structure-guided study of OXA-24 and OXA-48. ACS Chem Biol 10:591-600.

9. Outeda-García M, Arca-Suárez J, Lence E, Rodriguez-Coello A, Maceiras R, Blanco-Martin T, Guijarro-Sánchez P, Gonzalez-Pinto L, Alonso-Garcia I, García-Pose A, Muras A, Rodriguez-Pallares S, Lasarte-Monterrubio C, Gonzalez-Bello C, Vázquez-Ucha JC, Bou G, Beceiro A. 2025. Advancements in the fight against globally distributed OXA-48 carbapenemase: evaluating the new generation of carbapenemase inhibitors. Antimicrob Agents Chemother 69:e01614-24.

10. Bou G, Santillana E, Sheri A, Beceiro A, Sampson JM, Kalp M, Bethel CR, Distler AM, Drawz SM, Pagadala SRR, Van Den Akker F, Bonomo RA, Romero A, Buynak JD. 2010. Design, Synthesis and Crystal Structures of 6-Alkylidene-2’-Substituted Penicillanic Acid Sulfones as Potent Inhibitors of *Acinetobacter baumannii* OXA-24 Carbapenemase. J Am Chem Soc 132:13320-13331.

11. Winkler ML, Papp-Wallace KM, Hujer AM, Domitrovic TN, Hujer KM, Hurless KN, Tuohy M, Hall G, Bonomo RA. 2015. Unexpected challenges in treating multidrug-resistant gram-negative bacteria: Resistance to ceftazidime-avibactam in archived isolates of *Pseudomonas aeruginosa*. Antimicrob Agents Chemother 59:1020–1029.

12. Ehmann DE, Jahic H, Ross PL, Gu RF, Hu J, Durand-Réville TF, Lahiri S, Thresher J, Livchak S, Gao N, Palmer T, Walkup GK, Fisher SL. 2013. Kinetics of avibactam inhibition against Class A, C, and D β-lactamases. J Biol Chem 288:27960-71.

13. Papp-Wallace KM, Winkler ML, Taracila MA, Bonomo RA. 2015. Variants of β-lactamase KPC-2 that are resistant to inhibition by avibactam. Antimicrob Agents Chemother 59:3710–3717.

14. Tsivkovski R, Lomovskayaa O. 2020. Biochemical activity of vaborbactam. Antimicrob Agents Chemother 64:e01935-19.
